# Osteopathic empirical research: a bibliometric analysis from 1966 to 2018

**DOI:** 10.1186/s12906-021-03366-3

**Published:** 2021-07-07

**Authors:** Chantal Morin, Isabelle Gaboury

**Affiliations:** 1 School of Rehabilitation, Faculty of Medicine and Health Sciences, Université de Sherbrooke, Sherbrooke, Quebec Canada; 2 Department of Osteopathy, Centre Ostéopathique du Québec, Montreal, Quebec Canada; 3 Department of Family Medicine and Emergency, Faculty of Medicine and Health Sciences, Université de Sherbrooke, Sherbrooke, Quebec Canada

**Keywords:** Osteopathy, Osteopathic manipulation, Bibliometrics, Effects of intervention, Craniosacral, Visceral osteopathy, Randomized control trials, Case reports, Cohort, Pilot studies

## Abstract

**Background:**

Despite the increasing use of osteopathy, a manipulative complementary and alternative medicine therapy, in the general population, its efficacy continues to be debated. In this era of evidence-based practice, no studies have previously reviewed the scientific literature in the field to identify published knowledge, trends and gaps in empirical research. The aims of this bibliometric analysis are to describe characteristics of articles published on the efficacy of osteopathic interventions and to provide an overall portrait of their impacts in the scientific literature.

**Methods:**

A bibliometric analysis approach was used. Articles were identified with searches using a combination of relevant MeSH terms and indexing keywords about osteopathy and research designs in MEDLINE and CINAHL databases. The following indicators were extracted: country of primary author, year of publication, journals, impact factor of the journal, number of citations, research design, participants’ age group, system/body part addressed, primary outcome, indexing keywords and types of techniques.

**Results:**

A total of 389 articles met the inclusion criteria. The number of empirical studies doubled every 5 years, with the United States, Italy, Spain, and United Kingdom being the most productive countries. Twenty-three articles were cited over 100 times. Articles were published in 103 different indexed journals, but more than half (53.7%) of articles were published in one of three osteopathy-focused readership journals. Randomized control trials (*n* = 145; 37.3%) and case reports (*n* = 142; 36.5%) were the most common research designs. A total of 187 (48.1%) studies examined the effects of osteopathic interventions using a combination of techniques that belonged to two or all of the classic fields of osteopathic interventions (musculoskeletal, cranial, and visceral).

**Conclusion:**

The number of osteopathy empirical studies increased significantly from 1980 to 2014. The productivity appears to be very much in sync with practice development and innovations; however, the articles were mainly published in osteopathic journals targeting a limited, disciplinary-focused readership.

**Supplementary Information:**

The online version contains supplementary material available at 10.1186/s12906-021-03366-3.

## Background

Osteopathy is a hand-on complementary and alternative medicine (CAM) approach used to address pain and a variety of functional conditions. According to the World Health Organization benchmarks for training in osteopathy, osteopaths use a wide variety of therapeutic manual techniques to improve physiological function by addressing areas of tissue strain, stress, or dysfunction that may impede normal function of somatic system and related neural, vascular, and lymphatic elements [1]. Osteopathic practice aims principally to restore and maintain a person’s natural state of wellbeing that requires the neurological, musculoskeletal, circulatory and visceral structures to work in balance together [2]. Over the past 20 years, osteopathy has gained attention among the general population [3–8]. Despite the increasing use of osteopathy worldwide, its efficacy continues to be debated within both scientific and healthcare communities.

In the era of evidence-based practice, the lack of scientific evidence, especially from randomized controlled trials (RCT), is one of the most common criticisms against complementary and alternative medicine [9]. Limited evidence is also known to be a barrier to collaboration between the medical community and osteopaths [10]. A better scientific understanding of the mechanisms of action for osteopathic interventions, in particular unrelated to musculoskeletal problems, and a better dissemination of information about scientific evidence in osteopathy still requires attention [10]. Osteopaths recognize the importance of using evidence from research in clinical practice and to improve the quality of care [11, 12]. For them, evidence from osteopathic research can be useful in helping patients to understand the benefits of osteopathy for their health, helping general practitioners and other health professionals understand the role of osteopathy and providing scientific evidence for what osteopaths do [13]. However, clinical evidence is scare [11] and not always easily accessible [10].

Bibliometric analysis, including citation analysis, is a method to map, measure, monitor and study scientific outputs of a particular area of research [14]. It specifically aims to provide quantitative data on all research of a given field and offer a comprehensive perspective of trends, activity, achievement and influence of those research [15]. Previous bibliometric reports on overall CAM scientific productivity [16, 17], traditional Chinese medicine [18], yoga [19], and integrative and complementary and alternative medicine in oncology [20, 21] have help to establish future research priorities to support evidence-based practice. A bibliometric overview could help to inform researchers, practitioners, other healthcare professionals, policy makers and patients, and to clarify perceptions of scientific productivity. Considering the growth in popularity of osteopathy, it is important to describe and analyze the available publications reporting efficacy measured in trials. To date, there is no publication summarizing worldwide tends in empirical osteopathic publications.

The aims of this bibliometric analysis are to describe characteristics of articles published on the efficacy of osteopathic interventions and to provide an overall portrait of these publications as well as their impacts in the scientific literature.

## Methods

There are no ethical issues associated with bibliometric searches and analyses; this study did not require ethics committee approval.

### Search methods

Combinations of relevant MeSH terms and indexing keywords were searched for in MEDLINE and CINAHL databases from 1966 to 2018 inclusively. MeSH terms and indexing keywords were related to: 1) osteopathy, including approaches: musculoskeletal, visceral, cranial and craniosacral; and 2) all empirical research designs. An additional file shows this in more detail (see Additional file 1). Reference lists of systematic reviews were also searched manually for potential additional primary articles.

Three rounds of screening were conducted to determine eligibility for inclusion of articles. Two independent analysts screened titles and abstracts for inclusion and reviewed full texts of potentially eligible articles. Disagreements were resolved by consensus with the third analyst. All authors extracted data.

### Eligibility criteria

Papers were screened to include only empirical osteopathic studies. All studies that evaluated the effects of an osteopathic intervention were included. The primary intervention had to be described as an osteopathic technique or intervention, used in the context of an osteopathic approach or performed by an osteopath if the intervention could be performed by other types of manual therapists (e.g. spinal manipulation). No restrictions were placed on the duration of the intervention.

Randomized controlled trials (RCTs), pre-experimental (before-after) and quasi-experimental, cohort, case-control, case series and case reports designs published in English or French were included. Systematic reviews, animal model studies, surveys, inter rater validity studies, educational papers, descriptive studies about the use of osteopathy, implantation studies, letters, and opinion and comment publications were excluded. Studies on all types of participants were eligible without restriction as to age, sex or country.

### Data extraction and analysis

The following bibliometric indicators were extracted using a standardized data extraction form in Excel: country of primary author, year of publication, journals, impact factor of the journal, number of citations normalized for the year of publication (according to the Web of Science) and percentiles in the reference set (using the P100’ method) [22], research design, participants’ age group, system/body part addressed, primary outcome, indexing keywords and types of technique. The P100’ method consists in ranking the number of citations received by a paper, while ignoring the frequency information. This allows for a normalization of citations received and a more robust comparison of the number of citations in a given reference set, over time and across disciplines and journals. The P100’ differs from the P100 method by considering the frequency of papers with similar citation counts [22]. Descriptive statistics were used to summarize results. Journals in which papers had been published were categorized according to the discipline of their target readership. Median impact factors (when available) were then compared, by discipline, to median impact factors for journals associated with those disciplines (source: Journal Citation Reports, 2020). Co-authorship relation network was analyzed with the VOSviewer software, version 1.6.16 (www.vosviewer.com) using all authors who published at least two papers in the study database.

## Results

5029 articles were identified by the literature search and an additional 4 were manually identified, for a total of 5032. Duplicates (*n* = 57) and studies in a language other than English or French (*n* = 19) were removed. Five records dated between 1966 and 1980 were not accessible. After reading the titles and abstracts, 4471 articles were rejected as not meeting inclusion criteria. Of the remaining 481 full text reviewed, 92 were excluded either because the full article was not published (*n* = 19), the intervention was not specifically osteopathic (*n* = 12), or because the study did not describe the effects of the intervention (*n* = 61). The final analysis was carried out on 389 included articles. An additional file shows references of all articles included (see Additional file 2).

Publications of osteopathic scientific research originated from over 25 countries. Eleven countries published at least 4 articles, while the United Kingdom, Spain, Italy, and the United States were the most productive countries with a range of 22 to 221 publications each (Table 1). Three or fewer publications originated from Belgium, New Zealand, Poland, Turkey, South Korea, Israel, Norway, Netherlands, Russia, Sweden, China, Iceland, Iran and the Czech Republic. Articles were published in 103 different indexed journals. Only seven journals have published more than five articles on the efficacy of osteopathy (Table 1). Analysis of co-authorship of all authors who published at least 2 papers in collaboration reveals that most networks consisted in local teams of researchers with the exception of two groups who shared international collaboration links between scholars from the United Kingdom and Australia (representing 2 papers).

**Table 1:** Country of publication and journals

Characteristics^**a**^	n (%)
**Country of publication**	
United States	221 (56.8)
Italy	28 (7.2)
Spain	25 (6.4)
United Kingdom	22 (5.7)
France	16 (4.1)
Australia	14 (3.6)
Germany	12 (3.1)
Brazil	8 (2.1)
Canada	8 (2.1)
India	6 (1.5)
Austria	4 (1.0)
**Journals**	
Journal of American Osteopathic Association	111 (28.5)
American Academy of osteopathy Journal	58 (14.9)
International Journal of Osteopathic Medicine	40 (10.3)
Journal of Bodywork and movement therapies	22 (5.7)
Journal of alternative and complementary medicine	16 (4.1)
Complementary therapies in medicine	7 (1.8)
Manual therapy	7 (1.8)

^a^The list includes countries in which more than three papers had been published and journals in which more than five papers had been published

**Fig. 1:**
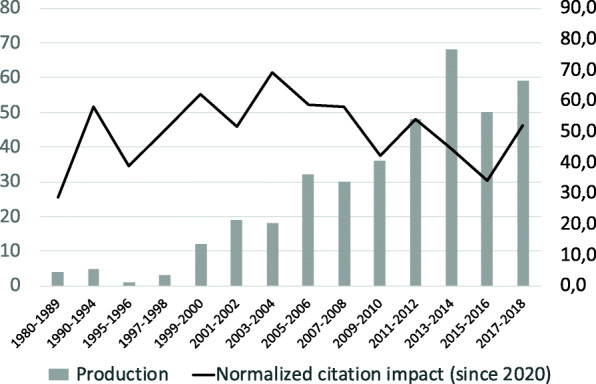
Research productivity (number of primary studies) and normalized citation impact

There was an exponential growth in the numbers of osteopathic research publications published between 1980 and 2014, with a roughly five-year doubling time (Fig. 1). From 2014 on, the annual number of research publications remained constant. The median frequency of citation was 10 (range 0 to 463). When adjusted for the number of years since publication, the median was 1 (range 0 to 19.2). The normalized citation impact appears to be slowly decreasing in the last decade. Twenty-three articles, all published prior to 2010, were cited more than 100 times. The top ten most cited articles were randomized control trials that originated from United States (*n* = 7), Australia (*n* = 2) and United-Kingdom (*n* = 1). Six (about low back pain and neck pain) were published in medical journals, three (one pediatric study on asthma and two adult study on lower limb) in osteopathic journals and one (low back pain) in physical therapy journal. They all examined musculoskeletal techniques, with one study also including a cranio-sacral technique, and another including a lymphatic intervention.

Impact factor of the journal used for publication ranged between 0.088 and 70.331 (median 1.466). A little less than half of the article (*n* = 159) were published in a journal with an impact factor. Normalized percentiles of citations and impact factors of retrieved articles (whenever available) showed low correlation (rho = 0.246, *p* < 0.001); i.e. papers published in high impact journals are not necessarily more cited than others. Table 2 presents the median impact factors by discipline and a comparison with median impact factors in the field (source: Journal Citation Reports, 2020). The median impact factors of osteopathic publications was generally lower than those of the corresponding disciplines, with the exception of obstetrics/gynecology, pediatrics, and rehabilitation. More than 3/4 (84.1%) of the osteopathic publications in these disciplines were RCTs (*n* = 31; 70.5%) or before/after experimental design (*n* = 6; 13.6%). Indeed, study design was found to be correlated with normalized citations (rho = 0.628, *p* < 0.001). RCTs were on average cited 4.5 times (normalized for years since publication); whereas case-controls, before-after, and cohorts were cited on average 3.3, 2.8, and 2.2 times respectively. Case series and case reports tended to have a smaller impact with 1.5 and 0.6 normalized citations on average.

**Table 2:** Median impact factors according to the discipline of the target readership

Discipline	Osteopathic publications selected; n; mean (min, max)	All topic literature; mean (min, max)
Arthritis/rheumatology	2; 1.909 (1.792, 2.025)	4.028 (0.316, 16.625)
Cancer	1; 2.773	7.209 (0.052, 292.278)
Gastroenterology	7; 2.337 (1.693, 3.424)	4.962 (0.658, 29.869)
General or internal medicine	102; 2.543 (0.103, 70.331)	3.457 (0.075, 74.699)
Obstetrics/gynecology	6; 2.884 (0.552, 5.642)	2.176 (0.139, 6.502)
Pediatrics	8; 2.540 (0.828, 5.485)	2.239 (0.156, 13.946)
Physiology	2; 3.59 (2.810, 4.371)	3.950 (0.111, 25.588)
Rehabilitation	30; 2.182 (0.088, 3.618)	1.568 (0.308, 3.657)

Table 3 summarizes the characteristics of the articles: study design, primary outcome, and population (Table 3). All research designs selected for this bibliometric analysis had been used. The analyses showed no significant increase in the proportion of RCTs over time (*p* = 0.963); however, osteopathy-oriented journals were more likely to publish case reports and case series.

**Table 3:** Study characteristics

Characteristics	n (%)
**Study design**	
Randomized control trial	145 (37.3)
Case report	142 (36.5)
Before-after	44 (11.3)
Cohort	27 (6.9)
Case series	20 (5.1)
Case-control	11 (2.8)
**Primary outcome**	
Function	151 (38.8)
Pain	150 (38.6)
Mobility	49 (12.6)
Psychosocial	26 (6.6)
Infectious	6 (1.5)
Cognitive	4 (1.0)
Physiological	3 (0.8)
**Population**	
Infants	24 (6.2)
1–6 years	17 (4.4)
7–18 years	24 (6.2)
Adults	306 (78.7)
65 years +	59 (15.2)
Pregnant women	10 (2.6)

Adults were most commonly studied. Study population sizes varied widely; for all types of designs, overall the median number of participants was 19.5 (25th, 75th percentiles = 1, 42 with a maximum of 1100 participants) but increased to 38 (25th, 75th percentiles = 22, 73) when single case reports were excluded. Function and pain were the most studied outcomes, accounting for over two-thirds of the primary endpoints. The keyword analysis did not yield any relevant information other than the populations, study designs, and conditions that emerge from the study.

The most popular techniques were myofascial techniques (nearly half of the studies), and muscle energy and High Velocity Low Amplitude (HVLA) techniques (one third of the studies) (Table 4). Treatments most commonly addressed the spine, the thoracic, abdominal and pelvic visceral systems, or limbs, followed by the cranial region (Table 4). A total of 82 (21.1%) articles reported the effect of a single osteopathic technique, 97 (24.9%) exclusively using musculoskeletal techniques, 15 (3.9%) cranial techniques and 5 (1.3%) visceral techniques. The remaining 187 (48.1%) articles studied the effects of an osteopathic intervention involving a combination of techniques that belonged to two or three of these fields of intervention (musculoskeletal, cranial and visceral techniques).

**Table 4:** Intervention characteristics

Characteristics	n (%)
**Techniques**^**a**^	
Musculoskeletal techniques	
Myofascial release	164 (42.2)
Muscle energy	127 (32.6)
High velocity low amplitude (HVLA)	118 (30.3)
Soft tissue	99 (25.4)
Strain counterstrain	75 (19.3)
Balanced ligamentous tension (BLT)	68 (17.5)
Mobilization	59 (15.2)
Articulatory	58 (14.9)
Diaphragm release	51 (13.1)
Rib raising	29 (7.5)
Facilitated positional release	29 (7.5)
Muscle inhibition	28 (7.2)
Still technique	16 (4.1)
Trigger points	8 (2.1)
General osteopathic treatment (GOT)	4 (1.0)
Spencer technique	3 (0.8)
Cranial techniques	
Suboccipital decompression	55 (14.1)
Cranial – nonspecific	47 (12.1)
Cranio-sacral and cranio-sacral therapy	35 (9.0)
Balanced membranous tension (BMT)	32 (8.2)
Compression of the fourth ventricle (CV4)	31 (8.0)
Sutural techniques	23 (5.9)
Sacral rocking, release or decompression	22 (5.7)
Spheno-basilar synchondrosis decompression	18 (4.6)
Mobilization temporal	16 (4.1)
Parietal and frontal lift	14 (3.6)
Dural tube traction	9 (2.3)
V-Spread	9 (2.3)
Venous sinus drainage	7 (1.8)
Eustachian tube drainage	5 (1.3)
Visceral techniques	
Lymphatic	37 (9.5)
Organ mobilization	34 (8.7)
Visceral fascial release	24 (6.2)
Visceral – nonspecific	19 (4.9)
Plexus techniques (mesenteric and coeliac plexus)	5 (1.3)
Chapman points	4 (1.0)
Recoil	2 (0.5)
Viscero-somatic reflex	1 (0.3)
Osteopathic manipulative techniques (OMT)	9 (2.3)
Non-classified techniques	5 (1.3)
**Body part/system targeted by the intervention**	
Vertebral	139 (35.8)
Cervical	51 (13.1)
Lumbar	36 (9.3)
Dorsal	26 (6.7)
Pelvis	25 (6.4)
All spine	1 (0.3)
Thoracic, abdominal and pelvic visceral tissues	95 (24.4)
Cardiorespiratory	33 (8.5)
Digestive	34 (8.7)
Urinary/gynecological	16 (4.1)
Lymphatic	12 (3.1)
Musculoskeletal other than vertebral	74 (19.0)
Upper body	27 (6.9)
Lower body	30 (7.7)
Not specified or general	17 (4.4)
Cranium or craniosacral	62 (15.9)
Cannot assess	13 (3.3)
Stress and mental health	6 (1.5)

^a^Number of studies using this technique, many techniques might be used in a same study

## Discussion

The total number of publications on the effects of osteopathy included, although small compared to the number of publications usually included in a bibliometric analysis for CAM in general [16, 17], is comparable to other bibliometric analysis for a particular CAM approach such as yoga (*n* = 486) [19]. This first bibliometric analysis of osteopathic research highlights a broad range of study designs, osteopathic approaches and outcomes. As shown in Fig. 1, empirical osteopathy-related trial publications doubled every 5 years in the first twenty-five years. This is more rapid than the overall scientific literature, where publications have been shown to double every 23 years [23]. This suggests a substantial increase overtime in documenting the efficacy of common interventions, and reflecting the rise of this profession around the world. Similar trends of accelerated development of scientific production followed by stabilization of rates of output are observed in the scientific productions for other complementary and integrative fields of medical practice [17, 18, 21]. Possible explanations for the increase in osteopathic scientific publication could be the growing number of osteopaths pursuing graduate studies in research, the need for a more evidence-based practice, as well as the development of initiatives to better structure, support, and stimulate osteopathic research capacity and international collaborations [24–27]. This particularly pertains to countries such as United States, United Kingdom and Australia, where regulation and university-based osteopathic education are well established [2]. For the same reasons, funding to conduct osteopathy-related research might be easier to obtain in those jurisdictions, thus leading to greater numbers of publications and facilitating international collaborations for those scholars. It is worth noting that more than half of the studies considered in this study originated from the United States, where osteopathy is considered to be a branch of biomedicine; whereas it is considered a complementary approach in all other jurisdictions [2].

According to our bibliometric analysis, research designs are polarized between low and high levels of evidence in the classical biomedical pyramid of evidence classification [28, 29]. Indeed, our analysis shows in Table 3 that other than case reports, the randomized control trial (many being characterized as pilot studies by authors) is the most common research design. The proportion of RCTs in osteopathic research is comparable to that reported in bibliometric analyses of other complementary and alternative medical practices [30]. Our research design observations are consistent with the conclusions of several systematic reviews of efficacy of osteopathic interventions, that indicate needs for more robust and larger studies of osteopathic efficacy [31–40]. Similar recommendations have emerged indicating needs for further research into mechanisms of action behind osteopathic interventions [41–44]. Such understanding of physiological mechanisms would also help to establish objective and measurable outcomes [45] and in turn support design of rigorous clinical trials [46].

The focus on RCT study design and systematic review, particularly in a relatively new and developing field, may limit understanding of the effects of a given approach. Understanding of the mechanisms of action and the impact of non-specific effects of a holistic approach using RCTs is a challenge in complementary and alternative medicine research since treatment is often complex and personalized [47, 48]. Thus, considering the evolution of the osteopathy body of knowledge, it would be strategic to encourage and take into account other research designs, including case studies and case reports. These research designs are the preferred strategies to investigate “how” and “why” questions about an intervention. Non-RCT studies may contribute useful descriptive data that are sensitive to the contexts within which the experiences take place [49] and can lead to a better understanding of the potential effects of osteopathic approach in order to design robust RCTs. Knowledge syntheses on the effects of osteopathy could therefore be more inclusive of other research designs, possibly presented as narrative reviews, until publication volume has increased and larger scale research is available.

The bibliometric analysis offers an innovative insight into the alignment of research efforts with clinical practice. The body regions and systems most often studied in osteopathic research (Table 4), i.e. the spine and pelvis, the thoracic, abdominal and pelvic visceral areas and systems, the extremities and finally the head and face region, are consistent with the regions and systems most frequently addressed in the clinical practice of osteopaths regardless of the healthcare system or regulation of osteopathy [4, 7, 50]. In addition, most of the often-cited scientific publications address the effects of osteopathic manual treatment on low back pain; the condition (along with cervical pain) recognized as the most common reason for osteopathic consultations [3, 4, 6–8, 51]. The musculoskeletal techniques most frequently reported in the scientific literature – namely myofascial release, muscle energy, HVLA, soft tissue and articulatory or mobilization techniques – are aligned with what are observed to be the preferred or most used techniques in practice [6–8, 11, 12, 50, 51]. The same is noted regarding less common techniques in what is termed the visceral osteopathic field, insofar as the two most frequently used techniques (lymphatic, and organ mobilization techniques) correspond to the proportion of the osteopaths using it in clinical practice [8]. In the cranial field of osteopathy, suboccipital decompression technique, non-specific cranial techniques, balanced membranous tension and cranio-sacral techniques were investigated in 43.4% of included studies; this information can help to document the contribution of cranial techniques used from a quarter [8, 12, 50, 51] to half [6, 7] of osteopaths in clinical practice. The very low percentage of studies using exclusively cranial (3.9%) or exclusively visceral (1.3%) techniques highlights the irrelevance of trying to study compartmentalized techniques of osteopathy. In fact, nearly half of the studies (48.1%) used techniques of at least two and sometimes all of the classical fields of osteopathy (musculoskeletal, visceral and cranial), which reflects the essence of this multi-system approach, and the concept that the person is a dynamic functional unit in which all parts are interrelated [1].

Finally, as shown in Table 1, more than half of the publications were published in the three classic journals for osteopathy: Journal of American osteopathic medicine (JAOA), American Academy of osteopathy Journal (AAO) and the International Journal of osteopathic medicine (IJOM). These journals target a limited, discipline-focused readership, but were more likely to support the publication of study designs at the lower end of the evidence continuum. The transfer of knowledge between research and practice therefore remains highly disciplinary and focused on practitioners with interested in research. Besides, publications in this bibliometric study have been published in relatively low impact factor journals, which might impede the capacity of scholars to reach the overall healthcare community. Broader and better dissemination of research results through open access publications and various media can improve the reach to both patients and practitioners [46], for a greater impact in the healthcare field, including medical researchers, health care insurers, government agencies, and the media; the media is noted to exert considerable influence over public opinion and, potentially over policy [52, 53].

## Limitations

Limitations of this bibliometric analysis include that the search strategy relied heavily on indexed journals content and a limited manual retrieval strategy. It is possible that studies disseminated through less accessible media (e.g., journals associated with the field that are not indexed and may not be peer-reviewed) were not retrieved using the combination of keywords chosen.

## Conclusion

This bibliometric analysis shows that publications about efficacy of osteopathy are relatively recent and have increased at a rapid pace over the last three decades. More than half of these publications are published in three osteopathic journals targeting a limited, disciplinary-focused readership. Our results highlight important needs for large efficacy and effectiveness trials, as well as study designs to further understanding of the mechanisms of action of the techniques being investigated. Finally, this bibliometric analysis can assist to identify osteopathy techniques and populations where further clinical research is required.

## Supplementary Information

**Additional file 1.** Final search strategy – Medline and Cinahl

**Additional file 2.** References of articles included

## Data Availability

Final search strategy and references of articles included in the bibliometric analysis are available as supplementing material. The datasets used and/or analysed during the current study are available from the corresponding author on reasonable request.

## Abbreviations

RCT: Randomized controlled trials
CAM: Complementary and alternative medicine
HVLA: High Velocity Low Amplitude
