# Supplementary material for: Osteopathic empirical research: a bibliometric analysis from 1966 to 2018
Source: BMC Complement Med Ther. 2021 Jul 7;21:196. doi: 10.1186/s12906-021-03366-3 (PMC8265137; doi:10.1186/s12906-021-03366-3)
Supplement: Supplementary file 1 — Additional file 1. Final search strategy – Medline and Cinahl [file 12906_2021_3366_MOESM1_ESM.docx]

**Additional file 1. Final search strategy – Medline and Cinahl**

| **#** | **Stratégie de recherche** |  |
| --- | --- | --- |
| 1 | (MH "Hospitals, Osteopathic") | Mesh and key words for osteopathy |
| 2 | (MH "Osteopathic Physicians") |  |
| 3 | (MH "Manipulation, Osteopathic") |  |
| 4 | (MH "Osteopathic Medicine") |  |
| 28 | TX cranial AND TX (osteopathic OR osteopath OR osteopathy)) |  |
| 29 | TX visceral AND TX (osteopathic OR osteopath OR osteopathy)) |  |
| 30 | TX (manual N3 therapy) AND TX (osteopathic OR osteopath OR osteopathy)) |  |
| 31 | TX musculoskeletal AND TX (osteopathic OR osteopath OR osteopathy)) |  |
| 33 | TX craniosacral |  |
| 34  35 | TX osteopathic N1 manipulative N1 treatment  TX osteopathic N1 manipulative N1 medicine |  |
| 5 | TX consecutive N3 case | Mesh and key words for research design |
| 6 | TX case |  |
| 7 | TX case N1 control |  |
| 8 | TX single N1 case |  |
| 9 | TX case N3 series |  |
| 10 | TX case N3 reports | Combinaison osteo  Combinaison design |
| 11 | TX cohort N3 stud* |  |
| 12 | TX longitudinal N3 stud* |  |
| 13 | TX comparative N3 stud* |  |
| 14 | TX evaluation N1 study* |  |
| 15 | TX observation N3 stud* |  |
| 16 | TX validation N1 stud* |  |
| 17 | TX clinical N3 trial |  |
| 18 | TX controlled N3 trial |  |
| 19 | TX controlled N3 stud* |  |
| 20 | TX intervention N3 stud* |  |
| 21 | TX effectiveness |  |
| 22 | TX quasi N1 experimental |  |
| 23 | TX pragmatic N3 trial |  |
| 24 | TX experimental N3 stud* |  |
| 25 | TX multicenter N3 stud* |  |
| 26 | TX pre test AND TX post test |  |
| 27 | TX pretest AND TX posttest |  |
| 32 | (MH "Case-Control Studies") OR (MH "Cohort Studies") OR (MH "Retrospective Studies") OR (MH "Prospective Studies") OR (MH "Controlled Before-After Studies") OR (MH "Cross-Sectional Studies") OR (MH "Clinical Trial, Phase IV") OR (MH "Clinical Trial, Phase III") OR (MH "Clinical Trial, Phase II") OR (MH "Clinical Trial, Phase I") OR (MH "Randomized Controlled Trial") OR (MH "Pragmatic Clinical Trial") OR (MH "Controlled Clinical Trial") OR (MH "Clinical Trial") OR (MH "Cross-Over Studies") OR (MH "Non-Randomized Controlled Trials as Topic") |  |
| 36 | TX pilot N1 stud* |  |
| 37 | TX pilot N1 trial |  |
|  |  |  |
| 38 | Osteo : S2 OR S3 OR S4 OR S28 OR S29 OR S30 OR S31 OR S33 OR S34 OR S35 |  |
| 39 | Devis : S5 OR S6 OR S7 OR S8 OR S9 OR S10 OR S11 OR S12 OR S13 OR S14 OR S15 OR S16 OR S17 OR S18 OR S19 OR S20 OR S21 OR S22 OR S23 OR S24 OR S25 OR S26 OR S27 OR S32 OR S36 OR S37 |  |
| 40 | Final : osteo AND devis : S38 AND S39 | Final results |
