# Supplementary material for: Osteopathic empirical research: a bibliometric analysis from 1966 to 2018
Source: BMC Complement Med Ther. 2021 Jul 7;21:196. doi: 10.1186/s12906-021-03366-3 (PMC8265137; doi:10.1186/s12906-021-03366-3)
Supplement: Supplementary file 2 — Additional file 2. References of articles included [file 12906_2021_3366_MOESM2_ESM.docx]

**Additional file 2. References of articles included**

1. Accorsi A, Lucci C, Di Mattia L, Granchelli C, Barlafante G, Fini F, et al. Effect of osteopathic manipulative therapy in the attentive performance of children with attention-deficit/hyperactivity disorder. The Journal Of The American Osteopathic Association. 2014;114(5):374-81.

2. Aderholt SF, S. The twins. Massage & Bodywork. 2006;22(1):80-2.

3. Alburquerque-Sendín F, Fernández-de-las-Peñas C, Santos-del-Rey M, Martín-Vallejo FJ. Immediate effects of bilateral manipulation of talocrural joints on standing stability in healthy subjects. Manual Therapy. 2009;14(1):75-80.

4. Alexander J. Resolution of New Daily Persistent Headache After Osteopathic Manipulative Treatment. The Journal Of The American Osteopathic Association. 2016;116(3):182-5.

5. Altınbilek T, Murat S, Yumuşakhuylu Y, İçağasıoğlu A. Osteopathic manipulative treatment improves function and relieves pain in knee osteoarthritis: A single-blind, randomized-controlled trial. Turkish Journal of Physical Medicine & Rehabilitation (2587-0823). 2018;64(2):114-20.

6. Amanda B, Manuela M, Antonia M, Claudio M, Gregorio B. Posturography measures and efficacy of different physical treatments in somatic tinnitus. The International Tinnitus Journal. 2010;16(1):44-50.

7. Andersen S, Fryer GA, McLaughlin P. The effect of talo-crural joint manipulation on range of motion at the ankle joint in subjects with a history of ankle injury. Australasian chiropractic & osteopathy: journal of the Chiropractic & Osteopathic College of Australasia. 2003;11(2):57-62.

8. Anderson RE, Seniscal C. A comparison of selected osteopathic treatment and relaxation for tension-type headaches. Headache. 2006;46(8):1273-80.

9. Andersson GB, Lucente T, Davis AM, Kappler RE, Lipton JA, Leurgans S. A comparison of osteopathic spinal manipulation with standard care for patients with low back pain. The New England Journal Of Medicine. 1999;341(19):1426-31.

10. Andicochea CT, Fulkerson J, Taylor BM, Portouw SJ. Manual Therapy for Chronic Low Back Pain in an F-5 Pilot. Military Medicine. 2015;180(10):e1132-e5.

11. Andreoli E, Troiani A, Tucci V, Barlafante G, Cerritelli F, Pizzolorusso G, et al. Osteopathic manipulative treatment of congenital talipes equinovarus: a case report. Journal Of Bodywork And Movement Therapies. 2014;18(1):4-10.

12. Apoznanski TE, Abu-Sbaih R, Terzella MJ, Yao S. Resolution of dacryostenosis after osteopathic manipulative treatment. The Journal Of The American Osteopathic Association. 2015;115(2):110-4.

13. Apoznanski TEF, Theodore B. Role of Osteopathic Manipulative Treatment in a Dynamic Case of Parkinson Disease and Levodopa-Induced Dyskinesia: A Case Report. AAO Journal. 2015;25(1):21-4.

14. Aquino A, Perini M, Cosmai S, Zanon S, Pisa V, Castagna C, et al. Osteopathic Manipulative Treatment Limits Chronic Constipation in a Child with Pitt-Hopkins Syndrome. Case Reports In Pediatrics. 2017;2017:5437830-.

15. Arab AMN, Mohammad Reza. The effect of cranial osteopathic manual therapy on somatic tinnitus in individuals without otic pathology: Two case reports with one year follow up. International Journal of Osteopathic Medicine. 2014;17(2):123-8.

16. Archambault-Ezenwa L, Brewer J, Markowski A. A comprehensive physical therapy approach including visceral manipulation after failed biofeedback therapy for constipation. Techniques in Coloproctology : Official Journal of SICCR, MSCP, ISCRS, ECTA, Colorectal Anal Group of Surgical Section of Chinese Medical Association, MSPFD. 2016;20(8):603-7.

17. Arienti C, Bosisio T, Ratti S, Miglioli R, Negrini S. Osteopathic Manipulative Treatment Effect on Pain Relief and Quality of Life in Oncology Geriatric Patients: A Nonrandomized Controlled Clinical Trial. Integrative Cancer Therapies. 2018;17(4):1163-71.

18. Arienti C, Daccò S, Piccolo I, Redaelli T. Osteopathic manipulative treatment is effective on pain control associated to spinal cord injury. Spinal Cord. 2011;49(4):515-9.

19. Arnadottir TS, Sigurdardottir AK. Is craniosacral therapy effective for migraine? Tested with HIT-6 Questionnaire. Complementary Therapies In Clinical Practice. 2013;19(1):11-4.

20. Attali T-V, Bouchoucha M, Benamouzig R. Treatment of refractory irritable bowel syndrome with visceral osteopathy: short-term and long-term results of a randomized trial. Journal Of Digestive Diseases. 2013;14(12):654-61.

21. Baker JPB, Charity D. Osteopathic manipulative treatment for Lyme disease-induced Bell's palsy: a case study. AAO Journal. 2013;23(1):12-5.

22. Ballantyne FF, G.; McLaughlin, P. The effect of muscle energy technique on hamstring extensibility: the mechanism of altered flexibility. Journal of Osteopathic Medicine. 2003;6(2):59-63.

23. Baltazar GA, Betler MP, Akella K, Khatri R, Asaro R, Chendrasekhar A. Effect of osteopathic manipulative treatment on incidence of postoperative ileus and hospital length of stay in general surgical patients. The Journal Of The American Osteopathic Association. 2013;113(3):204-9.

24. Bambridge A. Approaching myopia holistically: a case study and theoretical exploration. Journal Of Alternative And Complementary Medicine (New York, NY). 2002;8(3):371-7.

25. Barnes PL, Laboy F, 3rd, Noto-Bell L, Ferencz V, Nelson J, Kuchera ML. A comparative study of cervical hysteresis characteristics after various osteopathic manipulative treatment (OMT) modalities. Journal Of Bodywork And Movement Therapies. 2013;17(1):89-94.

26. Batt J, Neeki MM. Osteopathic manipulative treatment in tarsal somatic dysfunction: a case study. The Journal Of The American Osteopathic Association. 2013;113(11):857-61.

27. Beal MC, Vorro J, Johnston WL. Chronic cervical dysfunction: correlation of myoelectric findings with clinical progress. The Journal Of The American Osteopathic Association. 1989;89(7):891-900.

28. Belcastro MR, Backes CR, Chila AG. Bronchiolitis: a pilot study of osteopathic manipulative treatment, bronchodilators, and other therapy. The Journal Of The American Osteopathic Association. 1984;83(9):672-6.

29. Belvaux A, Bouchoucha M, Benamouzig R. Osteopathic management of chronic constipation in women patients. Results of a pilot study. Clinics And Research In Hepatology And Gastroenterology. 2017;41(5):602-11.

30. Bennett S, Macfarlane C, Vaughan B. The Use of Osteopathic Manual Therapy and Rehabilitation for Subacromial Impingement Syndrome: A Case Report. Explore: The Journal of Science & Healing. 2017;13(5):339-43.

31. Berkowitz MR. Application of osteopathy in the cranial field to successfully treat vertigo: a case series. AAO Journal. 2009;19(3):27-32.

32. Berkowitz MR. Holistic osteopathic approach reveals unusual etiology for vertigo with co-morbid headache: a case report. AAO Journal. 2010;20(1):33-5.

33. Berkowitz MR. Application of osteopathic manipulative treatment to a patient with unremitting chest pain and shortness of breath undergoing “Rule-Out Myocardial Infarction” protocol for one week. International Journal of Osteopathic Medicine. 2012;15(2):73-7.

34. Berkowitz MR. Application of osteopathy in the cranial field to treat left superior homonymous hemianopsia. International Journal of Osteopathic Medicine. 2014;17(2):119-22.

35. Berkowitz MR. Osteopathic approach to treating a patient with congenital infantile torticollis reveals unusual presentation of absence of concomitant cranial base strain pattern: A case report. International Journal of Osteopathic Medicine. 2017;25:46-8.

36. Berkowitz MR. Osteopathic approach to treating a patient with new onset low back pain following injury reveals unusual presentation of bilateral and symmetric symptoms: A case report. International Journal of Osteopathic Medicine. 2017;24:48-50.

37. Bialosky JE, Bishop MD, Robinson ME, Zeppieri G, Jr., George SZ. Spinal manipulative therapy has an immediate effect on thermal pain sensitivity in people with low back pain: a randomized controlled trial. Physical Therapy. 2009;89(12):1292-303.

38. Białoszewski D, Bebelski M, Lewandowska M, Słupik A. Utility of craniosacral therapy in treatment of patients with non-specific low back pain. Preliminary report. Ortopedia, Traumatologia, Rehabilitacja. 2014;16(6):605-15.

39. BoÃ«t CF, Sophie; Marsault, Julie; Toublan, DÃ©borah; Valot, Marie-Eve; Cheval, Arthur; Amyot d'Inville, Gonzague; Niel, StÃ©phane; GuihÃ©neuc, Pierre; Guihard, Gilles. High-velocity low-amplitude thrust manipulation of the lumbar spine immediately modifies soleus T reflex in asymptomatic adults. International Journal of Osteopathic Medicine. 2013;16(3):131-42.

40. Bockenhauer SE, Julliard KN, Lo KS, Huang E, Sheth AM. Quantifiable effects of osteopathic manipulative techniques on patients with chronic asthma. The Journal Of The American Osteopathic Association. 2002;102(7):371-5.

41. Boesler D, Warner M, Alpers A, Finnerty EP, Kilmore MA. Efficacy of high-velocity low-amplitude manipulative technique in subjects with low-back pain during menstrual cramping. The Journal Of The American Osteopathic Association. 1993;93(2):203.

42. Bradshaw DS, K. OMT for post-mastectomy lymphedema and rib pain: case report. AAO Journal. 2006;16(4):21-4.

43. Bramati-Castellarin I, Patel VB, Drysdale IP. Repeat-measures longitudinal study evaluating behavioural and gastrointestinal symptoms in children with autism before, during and after visceral osteopathic technique (VOT). Journal Of Bodywork And Movement Therapies. 2016;20(3):461-70.

44. Branyon B. Healing hands: using osteopathic manipulative treatment to address visceral structures through somatovisceral reflexes: a case study in gastroesophageal reflux disease. AAO Journal. 2008;18(4):29-31.

45. Brolinson PG, Smolka M, Rogers M, Sukpraprut S, Goforth MW, Tilley G, et al. Precompetition manipulative treatment and performance among Virginia Tech athletes during 2 consecutive football seasons: a preliminary, retrospective report. The Journal Of The American Osteopathic Association. 2012;112(9):607-15.

46. Brugman RF, K.; Fryer, G. The effect of osteopathic treatment on chronic constipation -- a pilot study. International Journal of Osteopathic Medicine. 2010;13(1):17-23.

47. Brumm LF, Janiski C, Balawender JL, Feinstein A. Preventive osteopathic manipulative treatment and stress fracture incidence among collegiate cross-country athletes. The Journal Of The American Osteopathic Association. 2013;113(12):882-90.

48. Brumm LFC, D. P.; Nogle, S. E.; Johnson, S. M. Looking beyond the soft tissue: illustrative case studies of groin injuries. Athletic Therapy Today. 2001;6(4):24-64.

49. Burmeister DB, Sacco R, Rupp V. Management of benign paroxysmal positional vertigo with the canalith repositioning maneuver in the emergency department setting. The Journal Of The American Osteopathic Association. 2010;110(10):602-4.

50. Burnham T, Higgins DC, Burnham RS, Heath DM. Effectiveness of osteopathic manipulative treatment for carpal tunnel syndrome: a pilot project. The Journal Of The American Osteopathic Association. 2015;115(3):138-48.

51. Burns DK, Wells MR. Gross range of motion in the cervical spine: the effects of osteopathic muscle energy technique in asymptomatic subjects. The Journal Of The American Osteopathic Association. 2006;106(3):137-42.

52. Burton AK, Tillotson KM, Cleary J. Single-blind randomised controlled trial of chemonucleolysis and manipulation in the treatment of symptomatic lumbar disc herniation. European Spine Journal: Official Publication Of The European Spine Society, The European Spinal Deformity Society, And The European Section Of The Cervical Spine Research Society. 2000;9(3):202-7.

53. Cabrera-Martos I, Valenza MC, Valenza-Demet G, Benítez-Feliponi A, Robles-Vizcaíno C, Ruiz-Extremera A. Effects of manual therapy on treatment duration and motor development in infants with severe nonsynostotic plagiocephaly: a randomised controlled pilot study. Child's Nervous System. 2016;32(11):2211-7.

54. Calman J. Case study: Exhalation somatic dysfunction after multiple rib fractures and pneumothorax. AAO Journal. 2010;20(1):17-22.

55. Carpenter SW, A. Osteopathic manipulative treatment of low back pain during labor. AAO Journal. 2001;11(3):21-3.

56. Casanova-Méndez A, Oliva-Pascual-Vaca A, Rodriguez-Blanco C, Heredia-Rizo AM, Gogorza-Arroitaonandia K, Almazán-Campos G. Comparative short-term effects of two thoracic spinal manipulation techniques in subjects with chronic mechanical neck pain: a randomized controlled trial. Manual Therapy. 2014;19(4):331-7.

57. Cassidy CM. Commentary on terminology and therapeutic principles: challenges in classifying complementary and alternative medicine practices. Journal of alternative and complementary medicine (New York, NY). 2002;8(6):893-5.

58. Castillo I, Wolf K, Rakowsky A. Concussions and Osteopathic Manipulative Treatment: An Adolescent Case Presentation. The Journal Of The American Osteopathic Association. 2016;116(3):178-81.

59. Castro-Sánchez AM, Lara-Palomo IC, Matarán-Peñarrocha GA, Saavedra-Hernández M, Pérez-Mármol JM, Aguilar-Ferrándiz ME. Benefits of Craniosacral Therapy in Patients with Chronic Low Back Pain: A Randomized Controlled Trial. Journal of Alternative & Complementary Medicine. 2016;22(8):650-7.

60. Castro-Sánchez AM, Matarán-Peñarrocha GA, Sánchez-Labraca N, Quesada-Rubio JM, Granero-Molina J, Moreno-Lorenzo C. A randomized controlled trial investigating the effects of craniosacral therapy on pain and heart rate variability in fibromyalgia patients. Clinical Rehabilitation. 2011;25(1):25-35.

61. Cerritelli F, Carinci F, Pizzolorusso G, Turi P, Renzetti C, Pizzolorusso F, et al. Osteopathic manipulation as a complementary treatment for the prevention of cardiac complications: 12-Months follow-up of intima media and blood pressure on a cohort affected by hypertension. Journal Of Bodywork And Movement Therapies. 2011;15(1):68-74.

62. Cerritelli F, Ginevri L, Messi G, Caprari E, Di Vincenzo M, Renzetti C, et al. Clinical effectiveness of osteopathic treatment in chronic migraine: 3-Armed randomized controlled trial. Complementary Therapies In Medicine. 2015;23(2):149-56.

63. Cerritelli F, Verzella M, Barlafante G. Quality of life in patients referring to private osteopathic clinical practice: a prospective observational study. Complementary Therapies In Medicine. 2014;22(4):625-31.

64. Channell MK. Modified Muncie technique: osteopathic manipulation for eustachian tube dysfunction and illustrative report of case. The Journal Of The American Osteopathic Association. 2008;108(5):260-3.

65. Channell MK, Mueller LL, Hahn R. Management of chronic posttraumatic headache: a multidisciplinary approach. The Journal Of The American Osteopathic Association. 2009;109(9):509-13.

66. Chapello IA. A case history of recent problem breathing and special OMT for the heart. AAO Journal. 2009;19(1):23-6.

67. Chapello IAT, M. A. Unrelenting abdominal pain of elusive origin: a case study. AAO Journal. 2002;12(1):21-5.

68. Cheshire A, Polley M, Peters D, Ridge D. Is it feasible and effective to provide osteopathy and acupuncture for patients with musculoskeletal problems in a GP setting? A service evaluation. BMC Family Practice. 2011;12:49-.

69. Chin Aj Fau - Fischione AD, Fischione Ad Fau - Shilian R, Shilian R Fau - Walter LM, Walter Lm Fau - Ratay SM, Ratay Sm Fau - Bejanishvili TY, Bejanishvili Ty Fau - Wynbrandt JH, et al. Tolerance of Rib Raising Among Hospitalized Patients: A Pilot Study. (1945-1997 (Electronic)).

70. Chmielewski RP, Nicole; Capalbo, Gina. Osteopathic manipulative treatment of pes anserine bursitis using the triple technique: a case report. AAO Journal. 2013;23(1):34-8.

71. Chong AB, Murray R. Osteopathic approach to a patient with chronic fatigue syndrome. AAO Journal. 2011;21(2):37-47.

72. Chown MW, L.; Rush, M.; Allan, S.; Stott, D.; Archer, M. A prospective study of patients with chronic back pain randomised to group exercise, physiotherapy or osteopathy. Physiotherapy. 2008;94(1):21-8.

73. Clark BC, Goss DA, Jr., Walkowski S, Hoffman RL, Ross A, Thomas JS. Neurophysiologic effects of spinal manipulation in patients with chronic low back pain. BMC Musculoskeletal Disorders. 2011;12:170-.

74. Clark BC, Walkowski S, Conatser RR, Eland DC, Howell JN. Muscle functional magnetic resonance imaging and acute low back pain: a pilot study to characterize lumbar muscle activity asymmetries and examine the effects of osteopathic manipulative treatment. Osteopathic Medicine And Primary Care. 2009;3:7-.

75. Clark RC. The case of a patient with persistent urinary urgency. AAO Journal. 2005;15(1):20-2.

76. Clements BG, P.; McLaughlin, P. The amelioration of atlanto-axial rotation asymmetry using high velocity low amplitude manipulation: is the direction of thrust important? Journal of Osteopathic Medicine. 2001;4(1):8-14.

77. Cohen-Lewe A. Osteopathic manipulative treatment for colonic inertia. The Journal Of The American Osteopathic Association. 2013;113(3):216-20.

78. Collins CK. Physical therapy management of complex regional pain syndrome I in a 14-year-old patient using strain counterstrain: a case report. The Journal Of Manual & Manipulative Therapy. 2007;15(1):25-41.

79. Colonvega MH, K.; Minotti, D., II; Williams, S. A case report of osteopathic manipulative treatment in a 14 year-old girl with McCune-Albright syndrome. AAO Journal. 2009;19(3):21-4.

80. Cramer D, Miulli DE, Valcore JC, Taveau JW, Do N, Hutton DS, et al. Effect of pedal pump and thoracic pump techniques on intracranial pressure in patients with traumatic brain injuries. The Journal Of The American Osteopathic Association. 2010;110(4):232-8.

81. Crow TK, D. A myofascial trigger point on the skull: treatment improves peak flow values in acute asthma patients. AAO Journal. 2006;16(1):23-30.

82. Crow WT. Manipulative treatment for idiopathic impotence in a 24-year-old water polo player. International Journal of Osteopathic Medicine. 2006;9(2):66-71.

83. Cruser dA, Maurer D, Hensel K, Brown SK, White K, Stoll ST. A randomized, controlled trial of osteopathic manipulative treatment for acute low back pain in active duty military personnel. The Journal Of Manual & Manipulative Therapy. 2012;20(1):5-15.

84. Cuccia AM, Caradonna C, Annunziata V, Caradonna D. Osteopathic manual therapy versus conventional conservative therapy in the treatment of temporomandibular disorders: a randomized controlled trial. Journal Of Bodywork And Movement Therapies. 2010;14(2):179-84.

85. Curi ACC, Maior Alves AS, Silva JG. Cardiac autonomic response after cranial technique of the fourth ventricle (cv4) compression in systemic hypertensive subjects. Journal of bodywork and movement therapies. 2018;22(3):666-72.

86. Cutler MJ, Holland BS, Stupski BA, Gamber RG, Smith ML. Cranial manipulation can alter sleep latency and sympathetic nerve activity in humans: a pilot study. Journal Of Alternative And Complementary Medicine (New York, NY). 2005;11(1):103-8.

87. D'Ippolito M, Tramontano M, Buzzi MG. Effects of Osteopathic Manipulative Therapy on Pain and Mood Disorders in Patients With High-Frequency Migraine. The Journal Of The American Osteopathic Association. 2017;117(6):365-9.

88. da Silva RCV, de Sá CC, Pascual-Vaca ÁO, de Souza Fontes LH, Herbella Fernandes FAM, Dib RA, et al. Increase of lower esophageal sphincter pressure after osteopathic intervention on the diaphragm in patients with gastroesophageal reflux. Diseases Of The Esophagus: Official Journal Of The International Society For Diseases Of The Esophagus / ISDE. 2013;26(5):451-6.

89. Daraï C, Bendifallah S, Foulot H, Ballester M, Chabbert-Buffet N, Daraï E. [Impact of osteopathic manipulative therapy in patient with deep with colorectal endometriosis: A classification based on symptoms and quality of life]. Gynecologie, Obstetrique, Fertilite & Senologie. 2017;45(9):472-7.

90. Daraï C, Deboute O, Zacharopoulou C, Laas E, Canlorbe G, Belghiti J, et al. Impact of osteopathic manipulative therapy on quality of life of patients with deep infiltrating endometriosis with colorectal involvement: results of a pilot study. European Journal Of Obstetrics, Gynecology, And Reproductive Biology. 2015;188:70-3.

91. Dardzinski JA, Ostrov BE, Hamann LS. Myofascial pain unresponsive to standard treatment: successful use of a strain and counterstrain technique with physical therapy. Journal Of Clinical Rheumatology: Practical Reports On Rheumatic & Musculoskeletal Diseases. 2000;6(4):169-74.

92. de Oliveira RF, Liebano RE, Costa LdCM, Rissato LL, Costa LOP. Immediate effects of region-specific and non-region-specific spinal manipulative therapy in patients with chronic low back pain: a randomized controlled trial. Physical Therapy. 2013;93(6):748-56.

93. Dean AL. Osteopathic diagnosis and the treatment of sacral fracture: a case study. AAO Journal. 2012;22(4):12-4.

94. Dearing JH, F. An examination of pressure-pain thresholds (PPT's) at myofascial trigger points (MTrP's), following muscle energy technique or ischaemic compression treatment. Manual Therapy. 2008;13(1):87-8.

95. Degenhardt BF, Darmani NA, Johnson JC, Towns LC, Rhodes DCJ, Trinh C, et al. Role of osteopathic manipulative treatment in altering pain biomarkers: a pilot study. The Journal Of The American Osteopathic Association. 2007;107(9):387-400.

96. Degenhardt BF, Johnson JC, Gross SR, Hagan C, Lund G, Curry WJ. Preliminary findings on the use of osteopathic manipulative treatment: outcomes during the formation of the practice-based research network, DO-Touch.NET. The Journal Of The American Osteopathic Association. 2014;114(3):154-70.

97. Degenhardt BF, Kuchera ML. Osteopathic evaluation and manipulative treatment in reducing the morbidity of otitis media: a pilot study. The Journal Of The American Osteopathic Association. 2006;106(6):327-34.

98. Dey MV, Lucas; Zegarra-Parodi, Rafael. Osteopathic care of a patient with gallstones: A retrospective case study. International Journal of Osteopathic Medicine. 2013;16(1):e17-8.

99. Diaz-Mancha JA, Heredia-Rizo AM, Fernandez-Seguín LM, Albornoz-Cabello M. Visceral treatment and nutritional patterns in the management of low back pain: a case study. Journal Of Alternative And Complementary Medicine (New York, NY). 2014;20(8):661-2.

100. Dickey JL. Postoperative osteopathic manipulative management of median sternotomy patients. The Journal Of The American Osteopathic Association. 1989;89(10):1309.

101. DiFrancisco-Donoghue J, Apoznanski T, de Vries K, Jung M-K, Mancini J, Yao S. Osteopathic manipulation as a complementary approach to Parkinson's disease: A controlled pilot study. NeuroRehabilitation. 2017;40(1):145-51.

102. Diniz LR, Nesi J, Curi AC, Martins W. Qualitative evaluation of osteopathic manipulative therapy in a patient with gastroesophageal reflux disease: a brief report. The Journal Of The American Osteopathic Association. 2014;114(3):180-8.

103. Dowling DJ. Progressive inhibition of neuromuscular structures (PINS) technique. The Journal Of The American Osteopathic Association. 2000;100(5):285.

104. Dugailly P-MF, SÃ©bastien; Maroye, Laura; Evers, Luc; Klein, Paul; Feipel, VÃ©ronique. Effect of a general osteopathic treatment on body satisfaction, global self perception and anxiety: A randomized trial in asymptomatic female students. International Journal of Osteopathic Medicine. 2014;17(2):94-101.

105. Dugailly PM, Coucke A, Salem W, Feipel V. Assessment of cervical stiffness in axial rotation among chronic neck pain patients: A trial in the framework of a non-manipulative osteopathic management. Clinical Biomechanics (Bristol, Avon). 2018;53:65-71.

106. Duncan B, McDonough-Means S, Worden K, Schnyer R, Andrews J, Meaney FJ. Effectiveness of osteopathy in the cranial field and myofascial release versus acupuncture as complementary treatment for children with spastic cerebral palsy: a pilot study. The Journal Of The American Osteopathic Association. 2008;108(10):559-70.

107. Durrani MM, Jayme D.; Flaum, Theodore B. Larson Syndrome of Dysautonomia in Parkinson Disease Managed With Osteopathic Manipulative Treatment: A Case Report. AAO Journal. 2015;25(2):18-32.

108. Edwards DJ, Young H, Johnston R. The Immediate Effect of Therapeutic Touch and Deep Touch Pressure on Range of Motion, Interoceptive Accuracy and Heart Rate Variability: A Randomized Controlled Trial With Moderation Analysis. Frontiers In Integrative Neuroscience. 2018;12:41-.

109. Eisenhart AW, Gaeta TJ, Yens DP. Osteopathic manipulative treatment in the emergency department for patients with acute ankle injuries. The Journal Of The American Osteopathic Association. 2003;103(9):417-21.

110. Elden H, Östgaard H-C, Glantz A, Marciniak P, Linnér A-C, Olsén MF. Effects of craniosacral therapy as adjunct to standard treatment for pelvic girdle pain in pregnant women: a multicenter, single blind, randomized controlled trial. Acta Obstetricia Et Gynecologica Scandinavica. 2013;92(7):775-82.

111. Elliott JM, Jacobson EJ, Centeno CJ, Emerson PL. Cranial manipulation with possible neurovascular contact injury at the cerebello-pontine angle: a case report. Alternative Therapies In Health And Medicine. 2003;9(4):112.

112. Emerson SS, Speece AJ, 3rd. Manipulation of the coccyx with anesthesia for the management of coccydynia. The Journal Of The American Osteopathic Association. 2012;112(12):805-7.

113. Espí-López GV, Inglés M, Soliva-Cazabán I, Serra-Añó P, Enix D. Effect of the soft-tissue techniques in the quality of life in patients with Crohn's disease. Medicine. 2018;97(51).

114. Espí-López GV, López-Bueno L, Vicente-Herrero MT, Martinez-Arnau FM, Monzani L. Efficacy of manual therapy on anxiety and depression in patients with tension-type headache. A randomized controlled clinical trial. International Journal of Osteopathic Medicine. 2016;22:11-20.

115. Feehan J, Macfarlane C, Vaughan B. Conservative management of a traumatic meniscal injury utilising osteopathy and exercise rehabilitation: A case report. Complementary Therapies In Medicine. 2017;33:27-31.

116. Feely RA, Kapraun HE. Progressive Infantile Scoliosis Managed With Osteopathic Manipulative Treatment. The Journal Of The American Osteopathic Association. 2017;117(9):595-9.

117. Fernandes WVB, Blanco CR, Politti F, de Cordoba Lanza F, Lucareli PRG, Corrêa JCF. The effect of a six-week osteopathic visceral manipulation in patients with non-specific chronic low back pain and functional constipation: study protocol for a randomized controlled trial. Trials. 2018;19(1):151-.

118. Fernández-de-las-Peñas CD, C.; Miangolarra-Page, J. C. Immediate changes in radiographically determined lateral flexion range of motion following a single cervical HVLA manipulation in patients presenting with mechanical neck pain: a case series. International Journal of Osteopathic Medicine. 2005;8(4):139-45.

119. Fernández-de-las-Peñas CF-C, J.; Plaza Fernández, A.; Lomas-Vega, R.; Miangolarra-Page, J. C. Dorsal manipulation in whiplash injury treatment: a randomized controlled trial. Journal of Whiplash & Related Disorders. 2004;3(2):55-72.

120. Fernández-Pérez AM, Peralta-Ramírez MI, Pilat A, Moreno-Lorenzo C, Villaverde-Gutiérrez C, Arroyo-Morales M. Can myofascial techniques modify immunological parameters? Journal Of Alternative And Complementary Medicine (New York, NY). 2013;19(1):24-8.

121. Fernández-Pérez AM, Peralta-Ramírez MI, Pilat A, Villaverde C. Effects of myofascial induction techniques on physiologic and psychologic parameters: a randomized controlled trial. Journal Of Alternative And Complementary Medicine (New York, NY). 2008;14(7):807-11.

122. Fleming RKS, K. T. Low back pain in rowers. AAO Journal. 2010;20(2):27-8.

123. Fleming RKS, Karen T.; Blanke, Kent J.; Johnson, Jane C. The effect of osteopathic manipulative treatment on length of stay in posterolateral postthoracotomy patients: A retrospective case note study. International Journal of Osteopathic Medicine. 2015;18(2):88-96.

124. Florance B-M, Frin G, Dainese R, Nébot-Vivinus M-H, Marine Barjoan E, Marjoux S, et al. Osteopathy improves the severity of irritable bowel syndrome: a pilot randomized sham-controlled study. European Journal Of Gastroenterology & Hepatology. 2012;24(8):944-9.

125. Flowers R. Gentle persuasion: releasing developmental restrictions with light touch craniosacral therapy. Massage & Bodywork. 2006;22(1):78-84.

126. Fornari M, Carnevali L, Sgoifo A. Single Osteopathic Manipulative Therapy Session Dampens Acute Autonomic and Neuroendocrine Responses to Mental Stress in Healthy Male Participants. The Journal Of The American Osteopathic Association. 2017;117(9):559-67.

127. Fraix M. Osteopathic manipulative treatment and vertigo: a pilot study. PM & R: The Journal Of Injury, Function, And Rehabilitation. 2010;2(7):612-8.

128. Fraix M, Gordon A, Graham V, Hurwitz E, Seffinger MA. Use of the SMART Balance Master to quantify the effects of osteopathic manipulative treatment in patients with dizziness. The Journal Of The American Osteopathic Association. 2013;113(5):394-403.

129. Fraiz M. Osteopathic manual medicine for vertigo: review of literature, case report, and future research. AAO Journal. 2009;19(2):25-9.

130. Fryer GA, J.; Lamaro, J. The effect of osteopathic treatment on people with chronic and sub-chronic neck pain: a pilot study. International Journal of Osteopathic Medicine. 2005;8(2):41-8.

131. Galindez-Ibarbengoetxea X, Setuain I, Ramírez-Velez R, Andersen LL, González-Izal M, Jauregi A, et al. Immediate Effects of Osteopathic Treatment Versus Therapeutic Exercise on Patients With Chronic Cervical Pain. Alternative Therapies in Health & Medicine. 2017;23(7):238-46.

132. Galindez-Ibarbengoetxea X, Setuain I, Ramírez-Velez R, Andersen LL, González-Izal M, Jauregi A, et al. Short-term effects of manipulative treatment versus a therapeutic home exercise protocol for chronic cervical pain: A randomized clinical trial. Journal Of Back And Musculoskeletal Rehabilitation. 2018;31(1):133-45.

133. Gamber RG, Shores JH, Russo DP, Jimenez C, Rubin BR. Osteopathic manipulative treatment in conjunction with medication relieves pain associated with fibromyalgia syndrome: results of a randomized clinical pilot project. The Journal Of The American Osteopathic Association. 2002;102(6):321-5.

134. Gandolfi M, Geroin C, Valè N, Marchioretto F, Turrina A, Dimitrova E, et al. Does myofascial and trigger point treatment reduce pain and analgesic intake in patients undergoing onabotulinumtoxinA injection due to chronic intractable migraine? (1973-9095 (Electronic)).

135. Geisser ME, Wiggert EA, Haig AJ, Colwell MO. A randomized, controlled trial of manual therapy and specific adjuvant exercise for chronic low back pain. The Clinical Journal Of Pain. 2005;21(6):463-70.

136. Genese JS. Osteopathic manipulative treatment for facial numbness and pain after whiplash injury. The Journal Of The American Osteopathic Association. 2013;113(7):564-7.

137. Gesslbauer C, Vavti N, Keilani M, Mickel M, Crevenna R. Effectiveness of osteopathic manipulative treatment versus osteopathy in the cranial field in temporomandibular disorders – a pilot study. Disability & Rehabilitation. 2018;40(6):631-6.

138. Giles PD, Hensel KL, Pacchia CF, Smith ML. Suboccipital decompression enhances heart rate variability indices of cardiac control in healthy subjects. Journal Of Alternative And Complementary Medicine (New York, NY). 2013;19(2):92-6.

139. Gillespie BR. Case study in pediatric asthma: the corrective aspect of craniosacral fascial therapy. Explore (New York, NY). 2008;4(1):48-51.

140. Gillespie BR. Case study in attention-deficit/hyperactivity disorder: the corrective aspect of craniosacral fascial therapy. Explore (New York, NY). 2009;5(5):296-8.

141. Gilliss AC, Swanson RL, 2nd, Janora D, Venkataraman V. Use of osteopathic manipulative treatment to manage compensated trendelenburg gait caused by sacroiliac somatic dysfunction. The Journal Of The American Osteopathic Association. 2010;110(2):81-6.

142. Girsberger W, Bänziger U, Lingg G, Lothaller H, Endler P-C. Heart rate variability and the influence of craniosacral therapy on autonomous nervous system regulation in persons with subjective discomforts: a pilot study. Journal Of Integrative Medicine. 2014;12(3):156-61.

143. Goldstein FJ, Jeck S, Nicholas AS, Berman MJ, Lerario M. Preoperative intravenous morphine sulfate with postoperative osteopathic manipulative treatment reduces patient analgesic use after total abdominal hysterectomy. The Journal Of The American Osteopathic Association. 2005;105(6):273-9.

144. González-Álvarez FJV, Marie Carmen; Cabrera-Martos, Irene; Torres-Sánchez, Irene; Valenza-Demet, Gerald. Effects of a diaphragm stretching technique on pulmonary function in healthy participants: A randomized-controlled trial. International Journal of Osteopathic Medicine. 2015;18(1):5-12.

145. Goodkin MB, Bellew LJ. Osteopathic manipulative treatment for postural orthostatic tachycardia syndrome. The Journal Of The American Osteopathic Association. 2014;114(11):874-7.

146. Gordon CG, J. Self-hypnosis and osteopathic soft tissue manipulation with a ballet dancer. Contemporary Hypnosis (John Wiley & Sons, Inc). 2003;20(4):209-14.

147. Gough AK, Allen. Relieving depression in an elderly woman with osteopathic manipulative treatment: a case report. AAO Journal. 2011;21(2):50-2.

148. Goyal K, Goyal M, Narkeesh K, John Samuel A, Sharma S, Chatterjee S, et al. The effectiveness of osteopathic manipulative treatment in an abnormal uterine bleeding related pain and health related quality of life (HR-QoL) - A case report. Journal Of Bodywork And Movement Therapies. 2017;21(3):569-73.

149. Goyal M, Aggarwal A, Goyal K, Garg P. Effectiveness of Osteopathic Therapy in the Treatment of Oral Submucous Fibrosis. Contemporary Clinical Dentistry. 2017;8(1):145-7.

150. Goyal M, Goyal K, Bathla M, Kanimozhi D, Narkeesh D. Efficacy of Myofascial Unwinding and Myofascial Release Technique in a Patient with Somatic Symptoms - A Case Report. Indian journal of psychological medicine. 2017;39(2):199-201.

151. Goyal M, Goyal K, Narkeesh K, Samuel AJ, Arumugam N. Osteopathic manipulative treatment for post mastectomy lymphedema: A case report. International Journal of Osteopathic Medicine. 2017;26:49-52.

152. Goyal M, Goyal K, Narkeesh K, Samuel AJ, Arumugam N, Chatterjee S, et al. Efficacy of Osteopathic Manipulative Treatment Approach in the Patient with Pulmonary Fibrosis in Critical Care Outpatient Department. Indian Journal of Critical Care Medicine. 2017;21(7):469-72.

153. Gray RE, Kasper K. Osteopathic Manipulative Treatment as a Novel Way to Manage Postvasectomy Pain Syndrome. The Journal Of The American Osteopathic Association. 2018.

154. Grimaldi M. [Painful perineum in all its forms. Contribution of manual medicine and osteopathy. Clinical study]. Journal De Gynécologie, Obstétrique Et Biologie De La Reproduction. 2008;37(5):449-56.

155. Groisman SS, Luciano; Rocha, Natália; Hoff, Fabrícia; Rodrigues, Márcia Elisabeth; Ehlers, João Arthur; Diniz, Leonardo Rios. H-reflex responses to High-Velocity Low-Amplitude manipulation in asymptomatic adults. International Journal of Osteopathic Medicine. 2014;17(3):160-6.

156. Grondin F, Hall T. Changes in cervical movement impairment and pain following orofacial treatment in patients with chronic arthralgic temporomandibular disorder with pain: A prospective case series. Physiotherapy theory and practice. 2017;33(1):52-61.

157. Guernsey 3rd DT, Leder A, Yao S. Resolution of Concussion Symptoms After Osteopathic Manipulative Treatment: A Case Report. The Journal Of The American Osteopathic Association. 2016;116(3):e13-e7.

158. Gugel MR, Johnston WL. Osteopathic manipulative treatment of a 27-year-old man after anterior cruciate ligament reconstruction. The Journal Of The American Osteopathic Association. 2006;106(6):346-9.

159. Guiney PA, Chou R, Vianna A, Lovenheim J. Effects of osteopathic manipulative treatment on pediatric patients with asthma: a randomized controlled trial. The Journal Of The American Osteopathic Association. 2005;105(1):7-12.

160. Hachem NEH, Mohamad. Nouvelle technique passive d'ouverture de la trompe d'Eustache. Évaluation par tympanométrie de l'effet d'une nouvelle manipulation sur l'ouverture de la trompe d'Eustache chez les enfants de moins de six ans atteints d'une otite moyenne avec ou sans effusion. Kinesitherapie Revue. 2012(132):25-33.

161. Haiden N, Pimpel B, Kreissl A, Jilma B, Berger A. Does visceral osteopathic treatment accelerate meconium passage in very low birth weight infants?- A prospective randomized controlled trial. Plos One. 2015;10(4):e0123530-e.

162. Hall H, Cramer H, Sundberg T, Ward L, Adams J, Moore C, et al. The effectiveness of complementary manual therapies for pregnancy-related back and pelvic pain: A systematic review with meta-analysis. Medicine. 2016;95(38):e4723-e.

163. Haller H, Cramer H, Werner M, Dobos G. Treating the sequelae of postoperative meningioma and traumatic brain injury: a case of implementation of craniosacral therapy in integrative inpatient care. Journal Of Alternative And Complementary Medicine (New York, NY). 2015;21(2):110-2.

164. Haller H, Lauche R, Cramer H, Rampp T, Saha FJ, Ostermann T, et al. Craniosacral Therapy for the Treatment of Chronic Neck Pain: A Randomized Sham-controlled Trial. Clinical Journal of Pain. 2016;32(5):441-9.

165. Hamilton LB, C.; Fryer, G. The effects of high-velocity, low-amplitude manipulation and muscle energy technique on suboccipital tenderness. International Journal of Osteopathic Medicine. 2007;10(2/3):42-9.

166. Hanson GC, Jones B, Bacon CJ, Moran RW. Exploration of clinical changes following a novel mobilisation technique for treatment of chronic low back pain: A single cohort design. Journal Of Bodywork And Movement Therapies. 2016;20(3):571-8.

167. Hanten WPO, S. L.; Hodson, J. L.; Imler, V. L.; Knab, V. M.; Magee, J. L. The effectiveness of CV-4 and resting position techniques on subjects with tension-type headaches. Journal of Manual & Manipulative Therapy (Journal of Manual & Manipulative Therapy). 1999;7(2):64-70.

168. Hatter AW, S. Understanding and treating fatigue and gait instability in persons with multiple sclerosis: an osteopathic approach. AAO Journal. 2007;17(4):28-30.

169. Hayden C, Mullinger B. A preliminary assessment of the impact of cranial osteopathy for the relief of infantile colic. Complementary Therapies In Clinical Practice. 2006;12(2):83-90.

170. Heineman K. Osteopathic manipulative treatment in the management of biliary dyskinesia. The Journal Of The American Osteopathic Association. 2014;114(2):129-33.

171. Henderson AT, Fisher JF, Blair J, Shea C, Li TS, Bridges KG. Effects of rib raising on the autonomic nervous system: a pilot study using noninvasive biomarkers. The Journal Of The American Osteopathic Association. 2010;110(6):324-30.

172. Hensel KL, Buchanan S, Brown SK, Rodriguez M, Cruser dA. Pregnancy Research on Osteopathic Manipulation Optimizing Treatment Effects: the PROMOTE study. American Journal Of Obstetrics And Gynecology. 2015;212(1):108.e1-9.

173. Hensel KL, Pacchia CF, Smith ML. Acute improvement in hemodynamic control after osteopathic manipulative treatment in the third trimester of pregnancy. Complementary Therapies In Medicine. 2013;21(6):618-26.

174. Hensel KL, Roane BM, Chaphekar AV, Smith-Barbaro P. PROMOTE Study: Safety of Osteopathic Manipulative Treatment During the Third Trimester by Labor and Delivery Outcomes. The Journal Of The American Osteopathic Association. 2016;116(11):698-703.

175. Herbert B. Chronic pelvic pain. Alternative Therapies In Health And Medicine. 2010;16(1):28-33.

176. Hernandez M, Chowdhury R, Woods J, Cabrera J, Hardigan PC. Management of suppurative cervical lymphadenitis in a healthy 24-year-old man. The Journal Of The American Osteopathic Association. 2011;111(1):49-51.

177. Herzhaft-Le Roy J, Xhignesse M, Gaboury I. Efficacy of an Osteopathic Treatment Coupled With Lactation Consultations for Infants' Biomechanical Sucking Difficulties. Journal Of Human Lactation: Official Journal Of International Lactation Consultant Association. 2017;33(1):165-72.

178. Hidalgo B, Hall T, Berwart M, Biernaux E, Detrembleur C. The immediate effects of two manual therapy techniques on ankle musculoarticular stiffness and dorsiflexion range of motion in people with chronic ankle rigidity: A randomized clinical trial. Journal Of Back And Musculoskeletal Rehabilitation. 2018;31(3):515-24.

179. Hidalgo-Lozano A, Fernández-de-las-Peñas C, Díaz-Rodríguez L, González-Iglesias J, Palacios-Ceña D, Arroyo-Morales M. Changes in pain and pressure pain sensitivity after manual treatment of active trigger points in patients with unilateral shoulder impingement: a case series. Journal Of Bodywork And Movement Therapies. 2011;15(4):399-404.

180. Hirai PM, Thomson OP. T4 syndrome – A distinct theoretical concept or elusive clinical entity? A case report. Journal of Bodywork & Movement Therapies. 2016;20(4):722-7.

181. Hoffman KS, Hoffman LL. Effects of adding sacral base leveling to osteopathic manipulative treatment of back pain: a pilot study. The Journal Of The American Osteopathic Association. 1994;94(3):217.

182. Hoving JL, Koes BW, de Vet HCW, van der Windt DAWM, Assendelft WJJ, van Mameren H, et al. Manual therapy, physical therapy, or continued care by a general practitioner for patients with neck pain. A randomized, controlled trial. Annals Of Internal Medicine. 2002;136(10):713-22.

183. Howell JN, Cabell KS, Chila AG, Eland DC. Stretch reflex and Hoffmann reflex responses to osteopathic manipulative treatment in subjects with Achilles tendinitis. The Journal Of The American Osteopathic Association. 2006;106(9):537-45.

184. Huard Y. Gait disturbance in the elderly: contribution of an osteopathic treatment. AAO Journal. 2013;23(1):40-5.

185. Hubert D, Soubeiran L, Gourmelon F, Grenet D, Serreau R, Perrodeau E, et al. Impact of osteopathic treatment on pain in adult patients with cystic fibrosis--a pilot randomized controlled study. Plos One. 2014;9(7):e102465-e.

186. Hundscheid HWC, Pepels MJAE, Engels LGJB, Loffeld RJLF. Treatment of irritable bowel syndrome with osteopathy: results of a randomized controlled pilot study. Journal Of Gastroenterology And Hepatology. 2007;22(9):1394-8.

187. Hurrell JS, A. J.; Williams, S. F. Manipulation of adhesive capsulitis under anesthesia. AAO Journal. 2006;16(2):27-8.

188. Ignatowicz A, Berkowitz MR. Imaging Evidence Demonstrating Effectiveness of Osteopathic Visceral Manipulation Techniques in Treating Pseudo-Obstruction. AAO Journal. 2017;27(1):7-11.

189. Jackson KM, Steele TF, Dugan EP, Kukulka G, Blue W, Roberts A. Effect of lymphatic and splenic pump techniques on the antibody response to hepatitis B vaccine: a pilot study. The Journal Of The American Osteopathic Association. 1998;98(3):155-60.

190. Jacq O, Arnulf I, Similowski T, Attali V. Upper airway stabilization by osteopathic manipulation of the sphenopalatine ganglion versus sham manipulation in OSAS patients: a proof-of-concept, randomized, crossover, double-blind, controlled study. BMC Complementary And Alternative Medicine. 2017;17(1):546-.

191. Jakub Hubert S, Dariusz C. Assessment of the effects of manual techniques on neck pain. Advances in Rehabilitation [Internet]. 2018; 2018(2):[33-9 pp.].

192. Jardine WMG, Carol; Rutherford, Derek. The effect of osteopathic manual therapy on the vascular supply to the lower extremity in individuals with knee osteoarthritis: A randomized trial. International Journal of Osteopathic Medicine. 2012;15(4):125-33.

193. Jarski RW, Loniewski EG, Williams J, Bahu A, Shafinia S, Gibbs K, et al. The effectiveness of osteopathic manipulative treatment as complementary therapy following surgery: a prospective, match-controlled outcome study. Alternative Therapies In Health And Medicine. 2000;6(5):77-81.

194. Jones ALL, M. D. Osteopathic manipulative treatment in pregnancy and augmentation of labor: a case report. AAO Journal. 2008;18(1):27-9.

195. Joyce PC, C. Seminar on CranioSacral Therapy. The use of CranioSacral Therapy to treat gastroesophageal reflux in infants. Infants & Young Children: An Interdisciplinary Journal of Early Childhood Intervention. 1996;9(2):51-8.

196. Kant RB, Murray R. Osteopathic Manipulative Treatment of Pelvic Dysfunction in a Postpartum Patient with Co-Morbid Headaches: A Case Report. AAO Journal. 2014;24(1):8-11.

197. Kaprow MG, Sandhouse M. Refractory torticollis after a fall. The Journal Of The American Osteopathic Association. 2000;100(3):148-50.

198. Karason AB, Drysdale IP. Somatovisceral response following osteopathic HVLAT: a pilot study on the effect of unilateral lumbosacral high-velocity low-amplitude thrust technique on the cutaneous blood flow in the lower limb. Journal Of Manipulative And Physiological Therapeutics. 2003;26(4):220-5.

199. Kary DJ. The transversus thoracis muscle in humans: diagnosis and treatment of associated pathology: an osteopathic perspective. AAO Journal. 2009;19(4):21-30.

200. Kennard EJ, Lieberman J, Saaid A, Rolfe KJ. A Preliminary Comparison of Laryngeal Manipulation and Postural Treatment on Voice Quality in a Prospective Randomized Crossover Study. Journal Of Voice: Official Journal Of The Voice Foundation. 2015;29(6):751-4.

201. Kilgore T, Malia M, Di Giacinto B, Minter S, Samies J. Adjuvant Lymphatic Osteopathic Manipulative Treatment in Patients With Lower-Extremity Ulcers: Effects on Wound Healing and Edema. The Journal Of The American Osteopathic Association. 2018;118(12):798-805.

202. Kim BJA, JungHoon; Cho, HeeCheol; Kim, DongYun; Kim, TaeYeong; Yoon, BumChul. Rehabilitation with osteopathic manipulative treatment after lumbar disc surgery: A randomised, controlled pilot study. International Journal of Osteopathic Medicine. 2015;18(3):181-8.

203. Kim M-yC, Eun Hya; Lee, Jin-ho; Ha, In-Hyuk. The clinical observation of muscle energy techniques and ligamentous articular strain in 2 cases of cervical disc herniation with thoracic outlet syndrome. International Journal of Osteopathic Medicine. 2015;18(1):63-70.

204. King HH, Tettambel MA, Lockwood MD, Johnson KH, Arsenault DA, Quist R. Osteopathic manipulative treatment in prenatal care: a retrospective case control design study. The Journal Of The American Osteopathic Association. 2003;103(12):577-82.

205. Klein R, Bareis A, Schneider A, Linde K. Strain-counterstrain to treat restrictions of the mobility of the cervical spine in patients with neck pain: a sham-controlled randomized trial. Complementary Therapies In Medicine. 2013;21(1):1-7.

206. Klock GB. The impact of osteopathic manipulative medicine on inpatient outcomes. AAO Journal. 2002;12(1):33-8.

207. Knebl JA, Shores JH, Gamber RG, Gray WT, Herron KM. Improving functional ability in the elderly via the Spencer technique, an osteopathic manipulative treatment: a randomized, controlled trial. The Journal Of The American Osteopathic Association. 2002;102(7):387-96.

208. Kobesova AL, K. A case of a pathogenic active scar. Australasian Chiropractic & Osteopathy. 2000;9(1):17-9.

209. Kohns DJ, Fitch DS. Osteopathic approach to sacroiliac dysfunction in a patient with steroid myopathy: case report and literature review. The Journal Of The American Osteopathic Association. 2014;114(6):498-504.

210. Kolkhorst ABK, D. J. Dig on: case report. AAO Journal. 2010;20(3):30-1.

211. Korotkov K, Shelkov O, Shevtsov A, Mohov D, Paoletti S, Mirosnichenko D, et al. Stress reduction with osteopathy assessed with GDV electrophotonic imaging: effects of osteopathy treatment. Journal Of Alternative And Complementary Medicine (New York, NY). 2012;18(3):251-7.

212. Kozminski MK, T. OMT as an adjunct therapy for post-traumatic headache in U.S. soldiers: a case series. AAO Journal. 2009;19(2):23-4.

213. Kramp ME. Combined manual therapy techniques for the treatment of women with infertility: a case series. The Journal Of The American Osteopathic Association. 2012;112(10):680-4.

214. Krishna HS, Ivor DsP, Basheer K. B R, Vishnu S. Study to Findout the Efficacy of Osteopathic Manual Therapy in Chest Expansion in COPD Patients. Indian Journal of Physiotherapy & Occupational Therapy. 2018;12(4):113-9.

215. Kuchera ML. Applying osteopathic principles to formulate treatment for patients with chronic pain. The Journal Of The American Osteopathic Association. 2007;107(10 Suppl 6):ES28-ES38.

216. Kumar AS, S. T.; Hensel, K. Ehlers Danlos Syndrome: a case report. AAO Journal. 2007;17(2):26-8.

217. Lafave MRS, Bonnie. Pudendal nerve entrapment in a bareback rodeo cowboy: A case study. International Journal of Osteopathic Medicine. 2012;15(2):78-82.

218. Lalonde F. The runner's kidney: A case report. International Journal of Osteopathic Medicine. 2014;17(3):206-10.

219. Lancaster DG, Crow WT. Osteopathic manipulative treatment of a 26-year-old woman with Bell's palsy. The Journal Of The American Osteopathic Association. 2006;106(5):285-9.

220. Lavelle JM, McKeigue ME. Musculoskeletal dysfunction and drop foot: diagnosis and management using osteopathic manipulative medicine. The Journal Of The American Osteopathic Association. 2009;109(12):648-50.

221. Leach J. Osteopathic support for a survivor of gastric cancer: a case report. International Journal of Osteopathic Medicine. 2008;11(3):106-11.

222. Leaver AM, Maher CG, Herbert RD, Latimer J, McAuley JH, Jull G, et al. A randomized controlled trial comparing manipulation with mobilization for recent onset neck pain. Archives Of Physical Medicine And Rehabilitation. 2010;91(9):1313-8.

223. Lenehan KLF, G.; McLaughlin, P. The effect of muscle energy technique on gross trunk range of motion. Journal of Osteopathic Medicine. 2003;6(1):13-8.

224. Lerma CM, M.; Hruby, R. J. An osteopathic approach to visceral disease: a case of recurrent urinary tract infection. AAO Journal. 2008;18(2):30-3.

225. Lessard S, Gagnon I, Trottier N. Exploring the impact of osteopathic treatment on cranial asymmetries associated with nonsynostotic plagiocephaly in infants. Complementary Therapies In Clinical Practice. 2011;17(4):193-8.

226. Lewis DD, Summers GK. Osteopathic Manipulative Treatment for the Management of Adjacent Segment Pathology. The Journal Of The American Osteopathic Association. 2017;117(12):782-5.

227. Lewis DD, Summers GK. An Osteopathic Approach to Low Back Pain and Short Leg Syndrome in a Patient with Traumatic Brain Injury Following Motor Vehicle Crash: A Case Report. AAO Journal. 2018;28(3):12-7.

228. Licciardone JC, Aryal S. Clinical response and relapse in patients with chronic low back pain following osteopathic manual treatment: results from the OSTEOPATHIC Trial. Manual Therapy. 2014;19(6):541-8.

229. Licciardone JC, Buchanan S, Hensel KL, King HH, Fulda KG, Stoll ST. Osteopathic manipulative treatment of back pain and related symptoms during pregnancy: a randomized controlled trial. American Journal Of Obstetrics And Gynecology. 2010;202(1):43.e1-8.

230. Licciardone JC, Gatchel RJ, Aryal S. Recovery From Chronic Low Back Pain After Osteopathic Manipulative Treatment: A Randomized Controlled Trial. The Journal Of The American Osteopathic Association. 2016;116(3):144-55.

231. Licciardone JC, Kearns CM, Hodge LM, Minotti DE. Osteopathic manual treatment in patients with diabetes mellitus and comorbid chronic low back pain: subgroup results from the OSTEOPATHIC Trial. The Journal Of The American Osteopathic Association. 2013;113(6):468-78.

232. Licciardone JC, Minotti DE, Gatchel RJ, Kearns CM, Singh KP. Osteopathic manual treatment and ultrasound therapy for chronic low back pain: a randomized controlled trial. Annals Of Family Medicine. 2013;11(2):122-9.

233. Licciardone JC, Stoll ST, Fulda KG, Russo DP, Siu J, Winn W, et al. Osteopathic manipulative treatment for chronic low back pain: a randomized controlled trial. Spine. 2003;28(13):1355-62.

234. Lipton JAC, J. Daren. Relief of persistent jaw pain with the use of osteopathic manipulative medicine. AAO Journal. 2013;23(2):15-7.

235. Lipton JAM, P.; Martin, J. B.; Mizera, A. C.; Kappler, R.; Brooks, J. S.; Parr, C. Improved pain score outcomes achieved through the cooperative and cost-effective use of physical (osteopathic manipulative) medicine in the treatment of outpatient musculoskeletal complaints. AAO Journal. 2002;12(1):26-32.

236. Lipton JAN, M.; Drew, B.; McCarty, C. A case of right first rib somatic dysfunction diagnosed and treated. AAO Journal. 2004;14(1):24-31.

237. Lipton JAN, M. A case study of left adhesive capsulitis presumably resulting from previous treatment with protease inhibitors. AAO Journal. 2004;14(4):17-9.

238. Lohr C, Schmidt T. Turnout in Classical Dance: Is It Possible to Enhance the External Rotation of the Lower Limb by a Myofascial Manipulation? A Pilot Study. Journal of Dance Medicine & Science. 2017;21(4):168-78.

239. Lombardini R, Marchesi S, Collebrusco L, Vaudo G, Pasqualini L, Ciuffetti G, et al. The use of osteopathic manipulative treatment as adjuvant therapy in patients with peripheral arterial disease. Manual Therapy. 2009;14(4):439-43.

240. Lopez D, King HH, Knebl JA, Kosmopoulos V, Collins D, Patterson RM. Effects of comprehensive osteopathic manipulative treatment on balance in elderly patients: a pilot study. The Journal Of The American Osteopathic Association. 2011;111(6):382-8.

241. Lund GC, Edwards G, Medlin B, Keller D, Beck B, Carreiro JE. Osteopathic manipulative treatment for the treatment of hospitalized premature infants with nipple feeding dysfunction. The Journal Of The American Osteopathic Association. 2011;111(1):44-8.

242. MacDonald RP, Theo; Leach, Janine. A randomized controlled pilot trial of positional release manipulation (counterstrain) in the treatment of restless legs syndrome. International Musculoskeletal Medicine. 2011;33(1):21-5.

243. Maddali Bongi S, Signorini M, Bassetti M, Del Rosso A, Orlandi M, De Scisciolo G. A manual therapy intervention improves symptoms in patients with carpal tunnel syndrome: a pilot study. Rheumatology International. 2013;33(5):1233-41.

244. Markelz KAB, Janice Upton. Osteopathic Manipulative Treatment for Patient With Severe Nausea and Vomiting in Pregnancy: A Case Study. AAO Journal. 2015;25(1):13-24.

245. Marske C, Bernard N, Palacios A, Wheeler C, Preiss B, Brown M, et al. Fibromyalgia with Gabapentin and Osteopathic Manipulative Medicine: A Pilot Study. Journal of Alternative & Complementary Medicine. 2018;24(4):395-402.

246. Marszałek S, Niebudek-Bogusz E, Woźnicka E, Malińska J, Golusiński W, Śliwińska-Kowalska M. Assessment of the influence of osteopathic myofascial techniques on normalization of the vocal tract functions in patients with occupational dysphonia. International Journal Of Occupational Medicine And Environmental Health. 2012;25(3):225-35.

247. Martí-Salvador M, Hidalgo-Moreno L, Doménech-Fernández J, Lisón JF, Arguisuelas MD. Osteopathic Manipulative Treatment Including Specific Diaphragm Techniques Improves Pain and Disability in Chronic Nonspecific Low Back Pain: A Randomized Trial. Archives of Physical Medicine & Rehabilitation. 2018;99(9):1720-9.

248. Martínez-Ochoa MJ, González-Iglesias J, Ricard F, Oliva-Pascual-Vaca Á, Fernández-Domínguez JC, Morales-Asencio JM. Effectiveness of an Osteopathic Abdominal Manual Intervention in Pain Thresholds, Lumbopelvic Mobility, and Posture in Women with Chronic Functional Constipation. Journal of Alternative & Complementary Medicine. 2018;24(8):816-24.

249. Martingano D. Management of Cesarean Deliveries and Cesarean Scars With Osteopathic Manipulative Treatment: A Brief Report. The Journal Of The American Osteopathic Association. 2016;116(7):e22-e30.

250. Matarán-Peñarrocha GA, Castro-Sánchez AM, García GC, Moreno-Lorenzo C, Carreño TP, Zafra MDO. Influence of craniosacral therapy on anxiety, depression and quality of life in patients with fibromyalgia. Evidence-Based Complementary And Alternative Medicine: Ecam. 2011;2011:178769-.

251. McCallister A, Brown C, Smith M, Ettlinger H, Baltazar GA. Osteopathic Manipulative Treatment for Somatic Dysfunction After Acute Severe Traumatic Brain Injury. The Journal Of The American Osteopathic Association. 2016;116(12):810-5.

252. McClennen ERG, R. Acute Intermittent Porphyria mimic of Guillain-Barré syndrome: a case report with the use of osteopathic manipulation for management of pain. AAO Journal. 2005;15(1):29-32.

253. McCoss CA, Johnston R, Edwards DJ, Millward C. Preliminary evidence of Regional Interdependent Inhibition, using a ‘Diaphragm Release’ to specifically induce an immediate hypoalgesic effect in the cervical spine. Journal of Bodywork & Movement Therapies. 2017;21(2):362-74.

254. McPartland JM, Giuffrida A, King J, Skinner E, Scotter J, Musty RE. Cannabimimetic effects of osteopathic manipulative treatment. The Journal Of The American Osteopathic Association. 2005;105(6):283-91.

255. McReynolds TM, Sheridan BJ. Intramuscular ketorolac versus osteopathic manipulative treatment in the management of acute neck pain in the emergency department: a randomized clinical trial. The Journal Of The American Osteopathic Association. 2005;105(2):57-68.

256. McSweeney TP, Thomson OP, Johnston R. The immediate effects of sigmoid colon manipulation on pressure pain thresholds in the lumbar spine. Journal Of Bodywork And Movement Therapies. 2012;16(4):416-23.

257. Mehl-Madrona L, Kligler B, Silverman S, Lynton H, Merrell W. The impact of acupuncture and craniosacral therapy interventions on clinical outcomes in adults with asthma. Explore (New York, NY). 2007;3(1):28-36.

258. Méndez-Sánchez R, Alburquerque-Sendín F, Fernández-de-las-Peñas C, Barbero-Iglesias FJ, Sánchez-Sánchez C, Calvo-Arenillas JI, et al. Immediate effects of adding a sciatic nerve slider technique on lumbar and lower quadrant mobility in soccer players: a pilot study. Journal Of Alternative And Complementary Medicine (New York, NY). 2010;16(6):669-75.

259. Méndez-Sánchez R, González-Iglesias J, Sánchez-Sánchez JL, Puente-González AS. Immediate effects of bilateral sacroiliac joint manipulation on plantar pressure distribution in asymptomatic participants. Journal Of Alternative And Complementary Medicine (New York, NY). 2014;20(4):251-7.

260. Meyer PM, Gustowski SM. Osteopathic manipulative treatment to resolve head and neck pain after tooth extraction. The Journal Of The American Osteopathic Association. 2012;112(7):457-60.

261. Miana L, Bastos VHdV, Machado S, Arias-Carrión O, Nardi AE, Almeida L, et al. Changes in alpha band activity associated with application of the compression of fourth ventricular (CV-4) osteopathic procedure: a qEEG pilot study. Journal Of Bodywork And Movement Therapies. 2013;17(3):291-6.

262. Mills MV, Henley CE, Barnes LLB, Carreiro JE, Degenhardt BF. The use of osteopathic manipulative treatment as adjuvant therapy in children with recurrent acute otitis media. Archives Of Pediatrics & Adolescent Medicine. 2003;157(9):861-6.

263. Milnes KM, R. W. Physiological effects of a CV4 cranial osteopathic technique on autonomic nervous system function: a preliminary investigation. International Journal of Osteopathic Medicine. 2007;10(1):8-17.

264. Minarini G, Ford M, Esteves J. Immediate effect of T2, T5, T11 thoracic spine manipulation of asymptomatic patient on autonomic nervous system response: Single-blind, parallel-arm controlled-group experiment. International Journal of Osteopathic Medicine. 2018;30:12-7.

265. Molins-Cubero S, Rodríguez-Blanco C, Oliva-Pascual-Vaca A, Heredia-Rizo AM, Boscá-Gandía JJ, Ricard F. Changes in pain perception after pelvis manipulation in women with primary dysmenorrhea: a randomized controlled trial. Pain Medicine (Malden, Mass). 2014;15(9):1455-63.

266. Monaco A, Cozzolino V, Cattaneo R, Cutilli T, Spadaro A. Osteopathic manipulative treatment (OMT) effects on mandibular kinetics: kinesiographic study. European Journal Of Paediatric Dentistry: Official Journal Of European Academy Of Paediatric Dentistry. 2008;9(1):37-42.

267. Morris HDD, J. L. Management of peptic ulcer disease using osteopathic manipulation. AAO Journal. 2007;17(1):26-9.

268. Muehlen NR, Michaela; Schwerla, Florian. Osteopathic treatment of somatoform autonomic dysfunctions of the cardiovascular system: A randomized controlled trial. International Journal of Osteopathic Medicine. 2013;16(1):e3-4.

269. Müller T, Pietsch A. Comparison of gait training versus cranial osteopathy in patients with Parkinson's disease: a pilot study. Neurorehabilitation. 2013;32(1):135-40.

270. Nelson KE, Sergueef N, Glonek T. The effect of an alternative medical procedure upon low-frequency oscillations in cutaneous blood flow velocity. Journal Of Manipulative And Physiological Therapeutics. 2006;29(8):626-36.

271. Nelson KES, N.; Glonek, T. Cranial manipulation induces sequential changes in blood flow velocity on demand. AAO Journal. 2004;14(3):15-9.

272. Nemett DR, Fivush BA, Mathews R, Camirand N, Eldridge MA, Finney K, et al. A randomized controlled trial of the effectiveness of osteopathy-based manual physical therapy in treating pediatric dysfunctional voiding. Journal Of Pediatric Urology. 2008;4(2):100-6.

273. Niel-Asher SH, Suzanne; Bentley, Stuart; Reynolds, Jonathan. Adhesive capsulitis: Prospective observational multi-center study on the Niel-Asher technique (NAT). International Journal of Osteopathic Medicine. 2014;17(4):232-42.

274. Nobles T, Bach A, Boesler D. Case report of osteopathic treatment of insomnia and traumatic anhidrosis. International Journal of Osteopathic Medicine. 2016;21:58-61.

275. Noll DR. The short-term effect of a lymphatic pump protocol on blood cell counts in nursing home residents with limited mobility: a pilot study. The Journal Of The American Osteopathic Association. 2013;113(7):520-8.

276. Noll DR, Degenhardt BF, Johnson JC. Multicenter Osteopathic Pneumonia Study in the Elderly: Subgroup Analysis on Hospital Length of Stay, Ventilator-Dependent Respiratory Failure Rate, and In-hospital Mortality Rate. The Journal Of The American Osteopathic Association. 2016;116(9):574-87.

277. Noll DR, Degenhardt BF, Johnson JC, Burt SA. Immediate effects of osteopathic manipulative treatment in elderly patients with chronic obstructive pulmonary disease. The Journal Of The American Osteopathic Association. 2008;108(5):251-9.

278. Noll DR, Degenhardt BF, Morley TF, Blais FX, Hortos KA, Hensel K, et al. Efficacy of osteopathic manipulation as an adjunctive treatment for hospitalized patients with pneumonia: a randomized controlled trial. Osteopathic Medicine And Primary Care. 2010;4:2-.

279. Noll DR, Degenhardt BF, Stuart MK, Werden S, McGovern RJ, Johnson JC. The effect of osteopathic manipulative treatment on immune response to the influenza vaccine in nursing homes residents: a pilot study. Alternative Therapies In Health And Medicine. 2004;10(4):74-6.

280. Noll DR, Shores J, Bryman PN, Masterson EV. Adjunctive osteopathic manipulative treatment in the elderly hospitalized with pneumonia: a pilot study. The Journal Of The American Osteopathic Association. 1999;99(3):143.

281. Noll DR, Shores JH, Gamber RG, Herron KM, Swift J, Jr. Benefits of osteopathic manipulative treatment for hospitalized elderly patients with pneumonia. The Journal Of The American Osteopathic Association. 2000;100(12):776-82.

282. Nourbakhsh MR, Fearon FJ. The effect of oscillating-energy manual therapy on lateral epicondylitis: a randomized, placebo-control, double-blinded study. Journal Of Hand Therapy: Official Journal Of The American Society Of Hand Therapists. 2008;21(1):4-13.

283. Novoseltsev SVV, D. B. Biomechanical disorders in the patients with lumbar discal hernias and their osteopathic correction. AAO Journal. 2010;20(1):11-5.

284. O-Yurvati AH, Carnes MS, Clearfield MB, Stoll ST, McConathy WJ. Hemodynamic effects of osteopathic manipulative treatment immediately after coronary artery bypass graft surgery. The Journal Of The American Osteopathic Association. 2005;105(10):475-81.

285. O'Connor S, Durand M-J, Hudson M, Baron M, Gaudreault N. Effects of osteopathic manipulative treatment on hand function, disease symptoms and functional status in systemic sclerosis: a series of single-case studies in working women. International Journal of Osteopathic Medicine. 2016;22:21-32.

286. Olds KB, Murray R. Isolated calcaneofibular ligament injuries treated with osteopathic manipulative treatment: A case series. International Journal of Osteopathic Medicine. 2012;15(4):166-72.

287. Oleski SL, Smith GH, Crow WT. Radiographic evidence of cranial bone mobility. Cranio: The Journal Of Craniomandibular Practice. 2002;20(1):34-8.

288. Oliva Pascual-Vaca AnPTDOP, Punzano-Rodríguez RnPTDO, Escribá-Astaburuaga PPTDO, Fernández-Domínguez JCPTP, Ricard FoDOP, Franco-Sierra MAPTDOP, et al. Short-Term Changes in Algometry, Inclinometry, Stabilometry, and Urinary pH Analysis After a Thoracolumbar Junction Manipulation in Patients with Kidney Stones. The Journal of Alternative and Complementary Medicine. 2017;23(8):639-47.

289. Origo D, Tarantino AG, Nonis A, Vismara L. Osteopathic manipulative treatment in chronic coccydynia: A case series. Journal Of Bodywork And Movement Therapies. 2018;22(2):261-5.

290. Papa L, Amodio A, Biffi F, Mandara A. Impact of osteopathic therapy on proprioceptive balance and quality of life in patients with dizziness. Journal Of Bodywork And Movement Therapies. 2017;21(4):866-72.

291. Papa L, Mandara A, Bottali M, Gulisano V, Orfei S. A randomized control trial on the effectiveness of osteopathic manipulative treatment in reducing pain and improving the quality of life in elderly patients affected by osteoporosis. Clinical Cases In Mineral And Bone Metabolism: The Official Journal Of The Italian Society Of Osteoporosis, Mineral Metabolism, And Skeletal Diseases. 2012;9(3):179-83.

292. Parker J, Heinking KP, Kappler RE. Efficacy of osteopathic manipulative treatment for low back pain in euhydrated and hypohydrated conditions: a randomized crossover trial. The Journal Of The American Osteopathic Association. 2012;112(5):276-84.

293. Pasquarello G. OMT relieves jaw pain. AAO Journal. 2003;13(2):34-5.

294. Pasquarello GJ. Pelvic pain due to placement of the vaginal cuff after hysterectomy: case report and osteopathic manipulative approach to treatment. AAO Journal. 2006;16(4):11-7.

295. Patel KG, Sabini RC. Safety of Osteopathic Cranial Manipulative Medicine as an Adjunct to Conventional Postconcussion Symptom Management: A Pilot Study. The Journal Of The American Osteopathic Association. 2018;118(6):403-9.

296. Paul LB, Murray R. Osteopathic manipulative treatment for an unusual presentation of fibromyalgia: A case report demonstrating the effectiveness of disease guidelines. International Journal of Osteopathic Medicine. 2015;18(2):141-7.

297. Pedowitz RN. Use of osteopathic manipulative treatment for iliotibial band friction syndrome. The Journal Of The American Osteopathic Association. 2005;105(12):563-7.

298. Pellerin F, Papin-Richard E, Guihéneuc P, Niel S, Guihard G. Can osteopathic manipulative treatment modify the posture in elderly people? - a single-case study. Journal Of Bodywork And Movement Therapies. 2015;19(2):380-8.

399. Peña-Salinas M, Oliva-Pascual-Vaca Js, Heredia-Rizo AM, Rodriguez-Blanco Cs, Ricard Fo, Oliva-Pascual-Vaca An. No immediate changes on neural and muscular mechanosensitivity after first rib manipulation in subjects with cervical whiplash: A randomized controlled trial. Journal of Back and Musculoskeletal Rehabilitation. 2017;30(4):921-8.

300. Perrin RNR, Jim David; Pentreath, V.; Percy, David F. Muscle fatigue in chronic fatigue syndrome/myalgic encephalomyelitis (CFS/ME) and its response to a manual therapeutic approach: A pilot study. International Journal of Osteopathic Medicine. 2011;14(3):96-105.

301. Peters TM, Roderic; Leach, C. M. J. Counterstrain manipulation in the treatment of restless legs syndrome: A pilot single-blind randomized controlled trial; the CARL Trial. International Musculoskeletal Medicine. 2012;34(4):136-40.

302. Petree K, Bruner J. Postoperative singultus: an osteopathic approach. The Journal Of The American Osteopathic Association. 2015;115(3):166-8.

303. Philippi H, Faldum A, Schleupen A, Pabst B, Jung T, Bergmann H, et al. Infantile postural asymmetry and osteopathic treatment: a randomized therapeutic trial. Developmental Medicine And Child Neurology. 2006;48(1):5-9.

304. Piche T, Pishvaie D, Tirouvaziam D, Filippi J, Dainese R, Tonohouhan M, et al. Osteopathy decreases the severity of IBS-like symptoms associated with Crohn's disease in patients in remission. European Journal Of Gastroenterology & Hepatology. 2014;26(12):1392-8.

305. Pizzolorusso G, Turi P, Barlafante G, Cerritelli F, Renzetti C, Cozzolino V, et al. Effect of osteopathic manipulative treatment on gastrointestinal function and length of stay of preterm infants: an exploratory study. Chiropractic & manual therapies. 2011;19(1):15-709X-19-15.

306. Plotkin BJ, Rodos JJ, Kappler R, Schrage M, Freydl K, Hasegawa S, et al. Adjunctive osteopathic manipulative treatment in women with depression: a pilot study. The Journal Of The American Osteopathic Association. 2001;101(9):517-23.

307. Polidori G, Kinne M, Mereu T, Beaumont F, Kinne M. Medical Infrared Thermography in back pain osteopathic management. Complementary Therapies In Medicine. 2018;39:19-23.

308. Pratt-Harrington D. Galbreath technique: a manipulative treatment for otitis media revisited. The Journal Of The American Osteopathic Association. 2000;100(10):635-9.

309. Prinsen JK, Hensel KL, Snow RJ. OMT associated with reduced analgesic prescribing and fewer missed work days in patients with low back pain: an observational study. The Journal Of The American Osteopathic Association. 2014;114(2):90-8.

310. Probst P, Büchler E, Doerr-Harim C, Knebel P, Thiel B, Ulrich A, et al. Randomised controlled pilot trial on feasibility, safety and effectiveness of osteopathic MANipulative treatment following major abdominal surgery (OMANT pilot trial). International Journal of Osteopathic Medicine. 2016;20:31-40.

311. Przekop PDOPD, Przekop ADO, Haviland MGPD. Multimodal compared to pharmacologic treatments for chronic tension-type headache in adolescents. Journal of Bodywork & Movement Therapies. 2016;20(4):715-21.

312. Racca V, Bordoni B, Castiglioni P, Modica M, Ferratini M. Osteopathic Manipulative Treatment Improves Heart Surgery Outcomes: A Randomized Controlled Trial. The Annals Of Thoracic Surgery. 2017;104(1):145-52.

313. Radjieski JM, Lumley MA, Cantieri MS. Effect of osteopathic manipulative treatment of length of stay for pancreatitis: a randomized pilot study. The Journal Of The American Osteopathic Association. 1998;98(5):264-72.

314. Raith W, Marschik PB, Sommer C, Maurer-Fellbaum U, Amhofer C, Avian A, et al. General Movements in preterm infants undergoing craniosacral therapy: a randomised controlled pilot-trial. BMC Complementary & Alternative Medicine. 2016;16:1-9.

315. Rajaii RM, Cox GJ, Schneider RP. Role of osteopathic manipulative treatment in the management of stiff person syndrome. The Journal Of The American Osteopathic Association. 2015;115(6):394-8.

316. Rancont CM. Chronic psoas syndrome caused by the inappropriate use of a heel lift. The Journal Of The American Osteopathic Association. 2007;107(9):415-8.

317. Randall S. Osteopathy: helping pregnant women in pain. The Practising Midwife. 2014;17(5):38-41.

318. Raviv G, Shefi S, Nizani D, Achiron A. Effect of craniosacral therapy on lower urinary tract signs and symptoms in multiple sclerosis. Complementary Therapies In Clinical Practice. 2009;15(2):72-5.

319. Reifsnyder JW, Tettambel MA. Conservative approach to tardive dyskinesia-induced neck and upper back pain. The Journal Of The American Osteopathic Association. 2013;113(8):636-9.

320. Ribar JSC, Todd A. Cranial and Fascial Distortion Techniques Used as Complementary Treatments to Alleviate Migraine Headache: A Case Report. AAO Journal. 2015;25(3):23-30.

321. Richards DGM, D. L.; Mein, E. A.; Nelson, C. D. Osteopathic regulation of physiology. AAO Journal. 2001;11(3):34-8.

322. Richardson BS, Way BV, Speece AJ, 3rd. Osteopathic manipulative treatment in the management of notalgia paresthetica. The Journal Of The American Osteopathic Association. 2009;109(11):605-8.

323. Ridgeway VB, M. R. Somatic dysfunction following sigmoid colon resection for diverticulitis: a case report. AAO Journal. 2010;20(2):25-8.

324. Riot F-M, Goudet P, Mouraux J-P, Cougard P. [Levator ani syndrome, functional intestinal disorders and articular abnormalities of the pelvis, the place of osteopathic treatment]. Presse Médicale (Paris, France: 1983). 2004;33(13):852-7.

325. Rivera-Martinez S, Wells MR, Capobianco JD. A retrospective study of cranial strain patterns in patients with idiopathic Parkinson's disease. The Journal Of The American Osteopathic Association. 2002;102(8):417-22.

326. Rivers WE, Treffer KD, Glaros AG, Williams CL. Short-term hematologic and hemodynamic effects of osteopathic lymphatic techniques: a pilot crossover trial. The Journal Of The American Osteopathic Association. 2008;108(11):646-51.

327. Rolle G, Tremolizzo L, Somalvico F, Ferrarese C, Bressan LC. Pilot trial of osteopathic manipulative therapy for patients with frequent episodic tension-type headache. The Journal Of The American Osteopathic Association. 2014;114(9):678-85.

328. Rook JLA, A. M. Multidisciplinary approach to treatment in a 38-year-old female, restrained driver following injuries sustained in a rear-end collision. AAO Journal. 2006;16(1):33-5.

329. Ross BSJ, Virginia M. Osteopathic Manipulative Treatment in Vestibular Neuritis: A Case Report. AAO Journal. 2014;24(2):27-32.

330. Ross G, Macfarlane C, Vaughan B. Combined osteopathy and exercise management of Achilles tendinopathy in an athlete. The Journal Of Sports Medicine And Physical Fitness. 2018;58(1-2):106-12.

331. Ruffini N, D'Alessandro G, Mariani N, Pollastrelli A, Cardinali L, Cerritelli F. Variations of high frequency parameter of heart rate variability following osteopathic manipulative treatment in healthy subjects compared to control group and sham therapy: randomized controlled trial. Frontiers In Neuroscience. 2015;9:272-.

332. Saggio G, Docimo S, Pilc J, Norton J, Gilliar W. Impact of osteopathic manipulative treatment on secretory immunoglobulin a levels in a stressed population. The Journal Of The American Osteopathic Association. 2011;111(3):143-7.

333. Sampson S, Meng M, Schulte A, Trainor D, Montenegro R, Aufiero D. Management of Dupuytren contracture with ultrasound-guided lidocaine injection and needle aponeurotomy coupled with osteopathic manipulative treatment. The Journal Of The American Osteopathic Association. 2011;111(2):113-6.

334. Sandhouse ME, Shechtman D, Sorkin R, Drowos JL, Caban-Martinez AJ, 3rd, Patterson MM, et al. Effect of osteopathy in the cranial field on visual function--a pilot study. The Journal Of The American Osteopathic Association. 2010;110(4):239-43.

335. Schwerla F, Kaiser AK, Gietz R, Kastner R. Osteopathic treatment of patients with long-term sequelae of whiplash injury: effect on neck pain disability and quality of life. Journal Of Alternative And Complementary Medicine (New York, NY). 2013;19(6):543-9.

336. Schwerla FW, Petra; Rütz, Michaela; Resch, Karl-Ludwig. Osteopathic treatment in patients with primary dysmenorrhoea: A randomised controlled trial. International Journal of Osteopathic Medicine. 2014;17(4):222-31.

337. Sergueef N, Nelson KE, Glonek T. Palpatory diagnosis of plagiocephaly. Complementary Therapies In Clinical Practice. 2006;12(2):101-10.

338. Shah RB, Murray R. Osteopathic manipulative treatment of isolated chronic sphenoidal sinusitis in a post-sinus surgery patient: a case report. AAO Journal. 2011;21(1):24-7.

339. Shanahan LKT, Raines SGM, Coggins RL, Moore T, Carnes M, Griffin L. Osteopathic Manipulative Treatment in the Management of Isaacs Syndrome. The Journal Of The American Osteopathic Association. 2017;117(3):194-8.

340. Shi X, Rehrer S, Prajapati P, Stoll ST, Gamber RG, Downey HF. Effect of cranial osteopathic manipulative medicine on cerebral tissue oxygenation. The Journal Of The American Osteopathic Association. 2011;111(12):660-6.

341. Shprecher D. Sensory trick with metoclopramide-associated tardive tremor. BMJ Case Reports. 2012;2012.

342. Silva ACdO, Biasotto-Gonzalez DA, Oliveira FHM, Andrade AO, Gomes CAFdP, Lanza FdC, et al. Effect of Osteopathic Visceral Manipulation on Pain, Cervical Range of Motion, and Upper Trapezius Muscle Activity in Patients with Chronic Nonspecific Neck Pain and Functional Dyspepsia: A Randomized, Double-Blind, Placebo-Controlled Pilot Study. Evidence-Based Complementary And Alternative Medicine: Ecam. 2018;2018:4929271-.

343. Smallwood CRB, Connie J.; Cox, Michele S.; Berkowitz, Murray R. Osteopathic manipulative treatment (OMT) during labor facilitates a natural, drug-free childbirth for a primigravida patient: A case report. International Journal of Osteopathic Medicine. 2013;16(3):170-7.

344. Smilowicz A. An osteopathic approach to gastrointestinal disease: somatic clues for diagnosis and clinical challenges associated with Helicobacter pylori antibiotic resistance. The Journal Of The American Osteopathic Association. 2013;113(5):404-16.

345. Smith L, Berkowitz MR. Osteopathic approach to chronic constipation in Prader–Willi Syndrome: A case report. International Journal of Osteopathic Medicine. 2016;19:73-7.

346. Smith M, Fryer G. A comparison of two muscle energy techniques for increasing flexibility of the hamstring muscle group. Journal Of Bodywork And Movement Therapies. 2008;12(4):312-7.

347. Snider KT. The Use of Osteopathic Manipulative Treatment as Part of an Integrated Treatment for Infantile Colic: A Case Report. AAO Journal. 2016;26(2):15-33.

348. Snider KT, Snider EJ, Johnson JC, Hagan C, Schoenwald C. Preventative osteopathic manipulative treatment and the elderly nursing home resident: a pilot study. The Journal Of The American Osteopathic Association. 2012;112(8):489-501.

349. Sonberg MM, B.; Rajendran, D. Can osteopathy help women with a history of hypothyroidism and musculoskeletal complaints? Outcome of a preliminary, prospective, open investigation. International Journal of Osteopathic Medicine. 2010;13(1):11-6.

350. Stager WH. Osteopathic manipulative medicine and acupuncture combined: a retrospective case study to determine if order of treatment makes a difference in outcome for acute mechanical low back pain. AAO Journal. 2007;17(4):11-22.

351. Steele KM, Carreiro JE, Viola JH, Conte JA, Ridpath LC. Effect of osteopathic manipulative treatment on middle ear effusion following acute otitis media in young children: a pilot study. The Journal Of The American Osteopathic Association. 2014;114(6):436-47.

352. Stretanski MF, Kaiser G. Osteopathic philosophy and emergent treatment in acute respiratory failure. The Journal Of The American Osteopathic Association. 2001;101(8):447-9.

353. Sucher BM. Myofascial release of carpal tunnel syndrome. The Journal Of The American Osteopathic Association. 1993;93(1):92.

354. Sucher BM. Palpatory diagnosis and manipulative management of carpal tunnel syndrome. The Journal Of The American Osteopathic Association. 1994;94(8):647-63.

355. Sucher BM. Ultrasonography-guided osteopathic manipulative treatment for a patient with thoracic outlet syndrome. The Journal Of The American Osteopathic Association. 2011;111(9):543-7.

356. Summers J, Ludwig J, Kanze D. Pierre robin sequence in a neonate with suckling difficulty and weight loss. The Journal Of The American Osteopathic Association. 2014;114(9):727-31.

357. Swender DA, Thompson G, Schneider K, McCoy K, Patel A. Osteopathic manipulative treatment for inpatients with pulmonary exacerbations of cystic fibrosis: effects on spirometry findings and patient assessments of breathing, anxiety, and pain. The Journal Of The American Osteopathic Association. 2014;114(6):450-8.

358. Tamer S, Öz M, Ülger Ö. The effect of visceral osteopathic manual therapy applications on pain, quality of life and function in patients with chronic nonspecific low back pain. Journal Of Back And Musculoskeletal Rehabilitation. 2017;30(3):419-25.

359. Tarsuslu T, Bol H, Simşek IE, Toylan IE, Cam S. The effects of osteopathic treatment on constipation in children with cerebral palsy: a pilot study. Journal Of Manipulative And Physiological Therapeutics. 2009;32(8):648-53.

360. Teale C. Craniosacral Therapy: A Case Study. Journal of the Australian Traditional-Medicine Society. 2013;19(4):226-7.

361. Teten Snider K. The Use of Osteopathic Manipulative Treatment for Acute Dental Pain: A Case Report. AAO Journal. 2016;26(1):17-25.

362. Thomaz SR, Teixeira FA, de Lima ACGB, Cipriano Júnior G, Formiga MF, Cahalin LP. Osteopathic manual therapy in heart failure patients: A randomized clinical trial. Journal Of Bodywork And Movement Therapies. 2018;22(2):293-9.

363. Thomson OH, L.; Mansfield, H. The effects of high-velocity low-amplitude thrust manipulation and mobilisation techniques on pressure pain threshold in the lumbar spine. International Journal of Osteopathic Medicine. 2009;12(2):56-62.

364. Thoreson M. Case study: an osteopathic approach to management of a patient with Charcot-Marie Tooth syndrome type II. AAO Journal. 2009;19(2):31-3.

365. Tobis JS, Hoehler FK. Musculoskeletal manipulation in the treatment of low back pain. Bulletin Of The New York Academy Of Medicine. 1983;59(7):660-8.

366. Todd C. Successful outcome of musculoskeletal injury leads to a reduction in chronic fatigue: A case report. Journal of Bodywork & Movement Therapies. 2018;22(2):281-6.

367. Tozzi P, Bongiorno D, Vitturini C. Fascial release effects on patients with non-specific cervical or lumbar pain. Journal Of Bodywork And Movement Therapies. 2011;15(4):405-16.

368. Tozzi P, Bongiorno D, Vitturini C. Low back pain and kidney mobility: local osteopathic fascial manipulation decreases pain perception and improves renal mobility. Journal Of Bodywork And Movement Therapies. 2012;16(3):381-91.

369. Tufo A, Desai GJ, Cox WJ. Psoas syndrome: a frequently missed diagnosis. The Journal Of The American Osteopathic Association. 2012;112(8):522-8.

370. Van Ravenswaay VJ, Hain SJ, Grasso S, Shubrook JH. Effects of Osteopathic Manipulative Treatment on Diabetic Gastroparesis. The Journal Of The American Osteopathic Association. 2015;115(7):452-8.

371. Vismara L, Cimolin V, Galli M, Grugni G, Ancillao A, Capodaglio P. Osteopathic Manipulative Treatment improves gait pattern and posture in adult patients with Prader–Willi syndrome. International Journal of Osteopathic Medicine. 2016;19:35-43.

372. Vismara L, Cimolin V, Menegoni F, Zaina F, Galli M, Negrini S, et al. Osteopathic manipulative treatment in obese patients with chronic low back pain: a pilot study. Manual Therapy. 2012;17(5):451-5.

373. Voigt K, Liebnitzky J, Burmeister U, Sihvonen-Riemenschneider H, Beck M, Voigt R, et al. Efficacy of osteopathic manipulative treatment of female patients with migraine: results of a randomized controlled trial. Journal Of Alternative And Complementary Medicine (New York, NY). 2011;17(3):225-30.

374. Wahl RA, Aldous MB, Worden KA, Grant KL. Echinacea purpurea and osteopathic manipulative treatment in children with recurrent otitis media: a randomized controlled trial. BMC Complementary And Alternative Medicine. 2008;8:56-.

375. Walko EJ, Janouschek C. Effects of osteopathic manipulative treatment in patients with cervicothoracic pain: pilot study using thermography. The Journal Of The American Osteopathic Association. 1994;94(2):135-41.

376. Wax CM, Abend DS, Pearson PH. Chest pain and the role of somatic dysfunction. The Journal Of The American Osteopathic Association. 1997;97(6):347.

377. Wells MR, Giantinoto S, D'Agate D, Areman RD, Fazzini EA, Dowling D, et al. Standard osteopathic manipulative treatment acutely improves gait performance in patients with Parkinson's disease. The Journal Of The American Osteopathic Association. 1999;99(2):92-8.

378. Wetzler G, Roland M, Fryer-Dietz S, Dettmann-Ahern D. CranioSacral Therapy and Visceral Manipulation: A New Treatment Intervention for Concussion Recovery. Medical Acupuncture. 2017;29(4):239-48.

379. Wiegand S, Bianchi W, Quinn TA, Best M, Fotopoulos T. Osteopathic manipulative treatment for self-reported fatigue, stress, and depression in first-year osteopathic medical students. The Journal Of The American Osteopathic Association. 2015;115(2):84-93.

380. Wieting JM, Beal C, Roth GL, Gorbis S, Dillard L, Gilliland D, et al. The effect of osteopathic manipulative treatment on postoperative medical and functional recovery of coronary artery bypass graft patients. The Journal Of The American Osteopathic Association. 2013;113(5):384-93.

381. Williams NH, Wilkinson C, Russell I, Edwards RT, Hibbs R, Linck P, et al. Randomized osteopathic manipulation study (ROMANS): pragmatic trial for spinal pain in primary care. Family Practice. 2003;20(6):662-9.

382. Wong CK, Gidali A, Harris V. Deformity or dysfunction? Osteopathic manipulation of the idiopathic cavus foot: A clinical suggestion. North American Journal Of Sports Physical Therapy: NAJSPT. 2010;5(1):27-32.

383. Wyatt K, Edwards V, Franck L, Britten N, Creanor S, Maddick A, et al. Cranial osteopathy for children with cerebral palsy: a randomised controlled trial. Archives Of Disease In Childhood. 2011;96(6):505-12.

384. Wynne MM, Burns JM, Eland DC, Conatser RR, Howell JN. Effect of counterstrain on stretch reflexes, hoffmann reflexes, and clinical outcomes in subjects with plantar fasciitis. The Journal Of The American Osteopathic Association. 2006;106(9):547-56.

385. Yates HA, Vardy TC, Kuchera ML, Ripley BD, Johnson JC. Effects of osteopathic manipulative treatment and concentric and eccentric maximal-effort exercise on women with multiple sclerosis: a pilot study. The Journal Of The American Osteopathic Association. 2002;102(5):267-75.

386. Zaidi TW, S. The use of OMT in patients with osteoarthritis: case report. AAO Journal. 2006;16(4):25-7.

387. Zanotti E, Berardinelli P, Bizzarri C, Civardi A, Manstretta A, Rossetti S, et al. Osteopathic manipulative treatment effectiveness in severe chronic obstructive pulmonary disease: a pilot study. Complementary Therapies In Medicine. 2012;20(1-2):16-22.

388. Zegarra-Parodi R, Pazdernik VK, Roustit M, Park PYS, Degenhardt BF. Effects of pressure applied during standardized spinal mobilizations on peripheral skin blood flow: A randomised cross-over study. Manual Therapy. 2016;21:220-6.

389. Zhang W, Guo W, Zhao P, Zhou W, Wei J, Li X-D, et al. Therapeutic effects of Chinese osteopathy in patients with lumbar disc herniation. The American Journal Of Chinese Medicine. 2013;41(5):983-94.
